# Integrated Chromatin Accessibility and Transcriptome Landscapes of 5-Fluorouracil-Resistant Colon Cancer Cells

**DOI:** 10.3389/fcell.2022.838332

**Published:** 2022-02-17

**Authors:** Bishu Zhang, Jiewei Lin, Jiaqiang Zhang, Xuelong Wang, Xiaxing Deng

**Affiliations:** ^1^ Department of General Surgery, Pancreatic Disease Center, Ruijin Hospital, Shanghai Jiao Tong University School of Medicine, Shanghai, China; ^2^ Research Institute of Pancreatic Diseases, Shanghai Jiao Tong University School of Medicine, Shanghai, China; ^3^ State Key Laboratory of Oncogenes and Related Genes, Shanghai, China; ^4^ Institute of Translational Medicine, Shanghai Jiao Tong University, Shanghai, China; ^5^ CAS Key Laboratory of Computational Biology, Shanghai Institute of Nutrition and Health, University of Chinese Academy of Sciences, Chinese Academy of Sciences, Shanghai, China

**Keywords:** 5-FU resistance, chromatin accessibility, colon cancer, differentially accessible regions, transcription factors

## Abstract

**Background:** 5-Fluorouracil (5-FU) is one of the most effective and widely used chemotherapeutic drugs in the treatment of colon cancer, yet chemoresistance is a common feature of colon cancer treatment, resulting in poor prognosis and short survival. Dynamic reprogramming of chromatin accessibility is crucial for proper regulation of gene transcription associated with cancer drug resistance by providing the gene regulatory machinery with rapid access to the open genomic DNA.

**Methods:** Here, we explored the global chromatin accessibility and transcription changes by the assay for transposase-accessible chromatin using sequencing (ATAC-seq) in combination with transcriptome sequencing of both parental and 5-FU-resistant HCT15 cells, followed by integrative analysis to better understand the regulatory network underlying 5-FU resistance in colon cancer cells.

**Results:** A total of 3,175 differentially expressed mRNAs (DEGs), lncRNAs (DELs), and miRNAs (DEMs) related to 5-FU resistance were identified, including significantly upregulated *IL33*, H19, and miR-17-5p; the downregulated *AKR1B10*, LINC01012, and miR-125b-5p; and chromatin modifiers such as INO80C, HDAC6, and KDM5A. The construction of the ceRNA regulatory network revealed that H19, HOXA11-AS, and NEAT1 might function as ceRNAs associated with 5-FU resistance in HCT15 cells. Moreover, 9,868 differentially accessible regions (DARs) were obtained, which were positively (r = 0.58) correlated with their nearest DEGs and DELs. The upregulated genes related to 4,937 hyper-accessible regions were significantly enriched in signaling pathways of MAPK, FOX, and WNT, while the 4,931 hypo-accessible regions were considered to be involved in declined biosynthesis of amino acids and nucleotide sugars, signaling pathways of Notch, and HIF-1. Analyses of the DAR sequences revealed that besides the AP-1 family, the TF motifs of FOX and KLF family members were highly enriched in hyper- and hypo-accessible regions, respectively. Finally, we obtained several critical TFs and their potential targets associated with DARs and 5-FU resistance, including FOXA1 and KLF3.

**Conclusion:** These data provided clear insights and valuable resources for an improved understanding of the non-genetic landscape of 5-FU-resistant colon cancer cells based on chromatin accessibility and transcript levels, which allowed for genome-wide detection of TF binding sites, potential *cis*-regulatory elements and therapeutic targets.

## Introduction

Colon cancer, a type of cancer that starts in the colon, is the third leading cause of cancer-related deaths worldwide, showing higher morbidity due to its aggressive behavior, poor prognosis, and lack of targeted treatments (Dekker et al., 2019; Sung et al., 2021). Identification of the influencing factors and molecular mechanisms driving the progression and recurrence of colon cancer is vital for its prevention and treatment.

5-Fluorouracil (5-FU) is a kind of antimetabolite drug and is widely used in the treatment of several cancer types, especially for colorectal cancer (Vodenkova et al., 2020). Currently, surgery and chemotherapy are the two main treatment options for colon cancer, and chemotherapy is generally given after surgery or radiotherapy as an adjuvant treatment for patients with advanced colon cancer (Hu et al., 2016). 5-FU-based chemotherapy remains the gold standard of first-line treatment for colon cancer, which exerts its cytotoxicity on cancer cells *via* inhibition of thymidylate synthase (TS) and incorporation of its metabolites into DNA and RNA (Longley et al., 2003). Over the past four decades, a more detailed understanding of the mechanisms of 5-FU transport, activation, action, and catabolism have led to the development of strategies that increase its anti-cancer activity (Hu et al., 2016; Blondy et al., 2020; Raskov et al., 2020). Despite these advances, drug resistance after chemotherapy remains a major limitation to the clinical application of 5-FU (Vodenkova et al., 2020; Sethy and Kundu 2021). Overcoming 5-FU resistance would represent a major therapeutic advance.

A growing number of genes, non-coding RNA, epigenetic regulators, and signaling pathways related to 5-FU resistance in colon cancer have been identified, such as *MRP1*, *FOXM1*, *CTCF*, *S1PR2*, miR-106a-5p, and lncRNA NEAT1 (Xie et al., 2017; Liu et al., 2018; Lai et al., 2020; Zhang Y. H et al., 2020; Zhu et al., 2020), however, the epigenetic regulatory mechanism, particularly the role of chromatin-mediated regulation of gene expression, remains poorly understood. Therefore, exploring the regulatory DNA elements and the corresponding TF binding sites of critical genes associated with chromatin accessibility changes is imperative for elucidation of the mechanisms of acquired resistance to 5-FU in colon cancer cells (CCCs).

As active regulatory genomic regions are usually “accessible,” while the closed chromatin regions impede access for the machinery of transcription, genome-wide chromatin accessibility profiling can be used to map and characterize candidate regulatory elements and transcriptional regulators (Buenrostro et al., 2013; Klemm et al., 2019). Depending on its high accuracy, simplicity, and low input cell number requirement, ATAC-seq has been a widely applied assay used to identify open and closed regions of chromatin and localize epigenetic changes underlying diverse development and disease-associated transitions, both in bulk and at the single-cell level (Buenrostro et al., 2013; Daugherty et al., 2017; Li et al., 2017; Cao et al., 2018; Lareau et al., 2019).

In the present study, we characterized the landscapes and relationship of chromatin accessibility and transcript expression in the parental and 5-FU-resistant HCT15 cells, using ATAC-seq coupled with transcriptome sequencing (mRNA, lncRNA, and miRNA). The integrative analysis of differential accessible regions (DARs) uncovered the landscape of binding events, regulatory DNA sequences, predict TFs, and their targets that were likely responsible for acquired resistance to 5-FU in colon cancer cells.

## Materials and Methods

### Cell Lines and Cell Culture

The HCT15 and 5-fluorouracil-resistant HCT15 (HCT15-FR) cell lines were purchased from the iCell Bioscience Inc. (Shanghai, China) and cultured in RPMI-1640 medium (KEL Biotech, China) with 10% fetal bovine serum (KEL Biotech, China), 2 mM L-glutamine (Gibco, United States), 0.1 M sodium pyruvate (Gibco, United States), 50 units/mL penicillin, and 50 μg/ml streptomycin (Sangon Biotech, China). All cells were grown to 80% confluence in 10-cm culture dishes in a humidified atmosphere with 5% CO_2_ at 37°C, refreshing culture medium every 2–3 days. The parental HCT15 cells were grown in a drug-free medium, whereas the HCT15-FR cells were maintained in 5-fluorouracil (iCell Bioscience Inc., China) at a concentration of 20 μg/ml according to the company’s protocol. 5-fluorouracil was stored at a concentration of 20 mg/ml (1,000 ×) in DMSO (Sigma-Aldrich, United States) at -20°C. The parental HCT15 cells were cultured in parallel with the HCT15-FR cells for comparison, and the HCT15-FR cells were maintained in 5-fluorouracil-free medium for 3 days before any further experiments were performed.

### Cell Viability Assay

Cell viability was assessed using the MTT assays (Roche, Germany) according to the manufacturer’s instructions. In brief, cells were seeded into 96-well plates and then incubated and allowed to grow at 37°C in the cell culture incubator. After different treatments, the medium was replaced with 100 μL of MTT solution and incubated at 37°C. After 2 h, MTT was removed and MTT–formazan crystals were dissolved in DMSO (100 μL/well). The absorbance was measured at 570 nm using a Safier II fluorescence reader (Tecan, Switzerland). The 50% cell growth inhibitory values (IC_50_) were determined by non-linear regression analysis using GraphPad Prism software.

### Library Preparation for Long-Insert Strand-specific Transcriptome Sequencing

Cells were harvested, and total RNA was extracted using TRIzol reagent (Thermo Fisher, United States). In total, 3 μg RNA per sample was used as input material for the RNA sample preparations. RNA quantity was assessed with a Qubit 3.0 Fluorometer (Thermo Fisher, United States). The ribosomal RNA was removed by using an Epicentre Ribo-zeroTM rRNA Removal Kit (Epicentre, United States). Sequencing libraries were constructed using a NEBNext UltraTM Directional RNA Library Prep Kit for Illumina (NEB, United States) according to the manufacturer’s instructions, which could obtain mRNAs and lncRNAs simultaneously. An Agilent 2100 Bioanalyzer (Agilent Technologies, United States) was used to assess RNA integrity. The library fragments were purified with the AMPure XP system (Beckman, United States) to select cDNA fragments of preferentially 250–300 bp. All libraries were sequenced on the Illumina HiSeq X Ten platform (Novogene Biotech, China). No less than 40 million 150-bp paired-end reads were needed for each sample.

### Library Preparation for Small Non-Coding RNA Sequencing

In total, 3 μg total RNA per sample was used as input material for the small RNA library. NEBNext Multiplex Small RNA Library Prep Set for Illumina (NEB, United States) was used to generate the sequencing libraries following the manufacturer’s instructions. Last, PCR products corresponding to 140-160 bp (the length of small non-coding RNA plus the 3′ and 5′ adapters) were purified and recovered. Library quality was assessed using the Agilent Bioanalyzer 2100 system and then sequenced on an Illumina Hiseq 2500 platform (Novogene Biotech, China). As a result, 50-bp single-end and no less than 10 million reads were generated for each sample.

### Assay for Transposase-Accessible Chromatin With High-Throughput Sequencing (ATAC-Seq)

ATAC-seq libraries were performed as previously reported (Buenrostro et al., 2013) with the following modifications. In brief, for each sample, 10,000 fresh cells were spun at 500 × g at 4°C for 5 min, followed by a wash with 50 μL of cold PBS buffer (KEL Biotech, China). Then cells were lysed using 100 µL cold lysis buffer [10 mM Tris–HCl (pH 7.4), 10 mM NaCl, 3 mM MgCl_2_ (Sangon Biotech, China), and 0.1% IGEPAL CA-630 (Sigma-Aldrich, United States)] on ice for 10–15 min to prepare the nuclei, then centrifuged at 500 × g for 10 min at 4°C. Immediately following the nuclei preparation, after removing the supernatant, the nuclei pellet was resuspended in 50 μL of Tn5 transposition reaction mix (Vazyme Biotech, China) prepared in advance for 30 min at 37°C. Directly following transposition, DNA was purified using a QIAquick PCR Purification Kit (QIAGEN, Germany). Then PCR was performed to amplify library fragments for a total of 10–15 cycles under the following PCR conditions: 72°C for 3 min; 98°C for 30 s; and thermocycling at 98°C for 15 s, 60°C for 30 s, and 72°C for 1 min; following by 72°C 5 min. The PCR products were purified using the VAHTS DNA Clean Beads (Vazyme Biotech, China) according to the manufacturer’s protocol. DNA quantity was assessed using a Qubit 3.0 Fluorimeter (Invitrogen, United States). Libraries were sequenced on the Illumina NovaSeq 6000 System (Novogene Biotech, China) for about 20 million paired-end reads with a fragment length of 150 bp per sample.

### Transcriptome Sequencing Data Processing

The Illumina FASTQ output files of sequences (including mRNAs and lncRNAs) were assessed for quality control using FastQC (version 0.11.6). The sequences were then aligned to the *Homo sapiens* reference genome (hg19) using STAR (Dobin et al., 2013). For each sample, the SAMtools view -s option was used to generate a .bam file containing a similar number of sequencing reads. The mapped reads were converted to FPKM (fragments per kilobase million reads) by running Cuffdiff (Trapnell et al., 2012) to quantify mRNA and lncRNA expression levels. Differential expression genes (DEGs) and lncRNAs (DELs) were filtered for a *p*-value < 0.05, the absolute value of fold change (FC) > 1.5 and the average FPKM >3 in at least one of the two groups. For genome browser visualization, SAMtools (Li et al., 2009) was used to filter paired-end reads from mapped BAM files and converted into the BigWig format using “bamCoverage” script from deepTools 2.0 (Ramirez et al., 2014).

### Small Non-Coding RNA Sequencing Data Processing

For small RNA sequencing, raw reads were assessed for quality control using FastQC (version 0.11.6). The Illumina adapters from reads were trimmed using Trimmomatic v0.39 (Bolger et al., 2014), and low-quality reads were discarded. The trimmed reads were aligned to the hg19 genome assembly (GRCh37) and quantified using the STAR (Dobin et al., 2013). The SAMtools view -s option was used to obtain a .bam file containing a similar number of sequencing reads. Small non-coding RNAs including miRNA, piRNA, and tRNA were identified by SPAR webserver (Kuksa et al., 2018). Differential expression miRNAs (DEMs) were identified using the DESeq2 (Love et al., 2014) package, with an adjusted *p*-value < 0.05 and the absolute value of fold change (FC) > 1.5. The miRWalk database (Dweep et al., 2011) was used for the prediction of the target genes of the miRNAs.

### ATAC-Seq Data Processing

Raw ATAC-seq data were assessed for quality control using FastQC (version 0.11.6). All sequences were aligned to the human genome assembly (hg19) using Bowtie2 (Langmead and Salzberg 2012) with the following parameter -X 2000. Duplicate reads were removed using SAMtools (Li et al., 2009), and only non-duplicate and properly paired-end reads kept in the BAM files were used for the subsequent analysis. Picard tools v.2.9.4 (https://broadinstitute.github.io/picard) and “ggplot2” package (Wickham 2009) in R were used to calculate and plot the distribution of fragment sizes from paired-end sequencing. The module “callpeak” in MACS2 (version 2.1.2) (Zhang et al., 2008) was used to identify the peaks of chromatin-accessible regions with the parameters --extsize 200 -shift -100. After peak calling, the “annotatePeaks.pl” script in HOMER (v4.10) (Heinz et al., 2010) was performed to annotate the location of a given peak in terms of genomic features, which includes whether a peak is in the Promoter-TSS (from −1,000 bp to +100 bp of transcription start site), TTS (from −100 bp to +1,000 bp of transcription termination site), 5′ UTR, 3′ UTR, exon, intronic, or intergenic by default defined. For normalization and visualization, the filtered and sorted BAM files were converted to the BigWig format using the “bamCoverage” scripts in deepTools v2.0 (Ramirez et al., 2014) with -bs 20 --normalizeUsing RPKM, and the “computeMatrix,” “plotHeatmap,” and “plotProfile” functions were conducted to generate heatmaps and average profiles displaying ATAC-seq signals.

### Identification of Differentially Accessible Regions

To further detail the genome-wide changes in chromatin accessibility, deepTools (Ramirez et al., 2014) was used to compute the average normalized RPKM values for ATAC-seq peaks in each group. The chromatin states were defined as “hyper-accessible” (herein referred to as “hyper”), if the peaks of Tn5 transposase hypersensitive sites showed a > 3-fold change in HCT15-FR cells compared to HCT15 cells. The “hypo-accessible” chromatin regions (i.e., “hypo”) were inversely correlated with decreased ATAC-seq signals in HCT15-FR cells, which showed a fold change larger than 3 in HCT15 cells. For data visualization, ChIPseeker (Yu et al., 2015), “plotHeatmap,” and “plotProfile” (Ramirez et al., 2014) were used to generate the heatmaps and average profiles of regions expanded to ±1,500 bp surrounding the DAR center.

### Correlation Analysis of Differential Gene Expression and Chromatin Accessibility

The “annotatePeaks.pl” script in HOMER (v4.10) (Heinz et al., 2010) was executed in order to annotate the location of DARs to genomic features, and then the nearest mRNAs and lncRNAs of DARs (within 100 kb of the TSS) were overlapped with DEGs and DELs identified from transcriptome sequencing. Fold changes of DARs and their nearest DEGs and DELs were used to calculate Pearson’s correlation coefficient (PCC) and *p*-value by the “Hmisc” R package (https://hbiostat.org/R/Hmisc/). “VennDiagram” (Chen and Boutros 2011) and “ggplot2” (Wickham 2009) packages were used to generate Venn diagrams, box plots, and scatter plots.

### GO Function and KEGG Pathway Enrichment Analysis

Enrichment analysis of the GO term and KEGG pathway was acquired *via* the R package “clusterProfiler” (Yu et al., 2012) with the hypergeometric distribution test. Enriched GO terms and KEGG pathways with *p*-value < 0.05 were considered significant. Network analysis was performed to display links between significantly enriched KEGG pathways and related DEGs using Cytoscape version 3.9.0 (http://www.cytoscape.org/).

### Gene Set Enrichment Analysis

Gene set enrichment analysis was performed on DEG lists ranked by the log_2_(fold change) using “clusterProfiler” (Yu et al., 2012) with Hallmark and KEGG gene sets downloaded from MSigDB v7.4 (http://software.broadinstitute.org/gsea/msigdb) and WebGestalt (http://www.webgestalt.org) (Wang et al., 2013).

### Construction of the Competing Endogenous RNA Network

The LncSEA database (Chen et al., 2021) was used to predict target miRNAs of DELs. The miRWalk 2.0 (Dweep et al., 2011) was used to search mRNA targeted by DEMs based on the TargetScan (http://www.targetscan.org/vert_80), miRDB (http://mirdb.org), miRTarBase (https://mirtarbase.cuhk.edu.cn) databases, and only those targets predicted by these three mentioned databases were considered for further analysis. Finally, for integrated transcriptome and small RNA sequencing, the “ggalluvial” R package (Rosvall and Bergstrom 2010) was used to establish and visualize the lncRNA-miRN-mRNA ceRNA network on the foundation of the interactions between DELs and DEMs, and between DEMs and DEGs.

### Transcription Factor Binding Motif Enrichment and Occurrence Analysis

To examine the enrichment of known transcription factor (TF) binding motifs in DARs, BEDTools v2.28.0 (Quinlan 2014) was used to obtain the ATAC-seq peak summits located in hyper- or hypo-accessible regions, and then “findMotifsGenome.pl” in HOMER v4.10 (Heinz et al., 2010) was employed to find known motifs and TF binding sites surrounding peak summits with the option -size −200,200. HOMER v4.10 (Heinz et al., 2010) was also used to identify potential target DEGs and to calculate the occurrence probability of a certain TF within a 1-Kb region flanking peak summits (from −500 to +500 bp). After motif enrichment analysis, the findMotifsGenome.pl script was used to find the binding sites of a certain TF (TFBSs) according to the tutorial instructions and then extract information about the genomic location of TFBSs from the output file. The annotation of the obtained TFBSs was performed *via* annotatePeaks.pl function to get the nearest genes of TFBSs. We defined the potential target DEGs of a certain TF when the absolute distance between transcription start sites (TSS) and TFBS is less than 3 Kb.

### TF Footprinting Analysis

To determine the TF footprints associated with the DARs, the BAM files for all biological replicates were merged to generate a composite BAM file using “SAMtools merge,” and HINT v0.13.1 (Gusmao et al., 2016) was used with the following parameters: rgt-hint function footprinting with the options --organism hg19 --paired-end --atac-seq to identify TF footprints in each sample; rgt-motif analysis function matching to match footprints to known TFs; and rgt-hint function differential with the default parameters. Differential HINT footprinting was performed for all DARs.

### Protein–Protein Interaction Network and Correlation Analysis

To obtain information regarding predicted and experimental interactions of differentially expressed transcription factors (DETFs) associated with DARs, we constructed a PPI network using Search Tool for the Retrieval of Interacting Genes (STRING) (Szklarczyk et al., 2017). Subnetwork modules were identified by the MCL (Markov Cluster Algorithm) with the default inflation parameter 3.0. The co-expression scores between proteins were calculated based on gene expression patterns, and protein co-regulation derived from ProteomeHD (Kustatscher et al., 2019).

### Statistical Analysis and Data Visualization

R platform (https://www.R-project.org/) was used to generate figures and to perform statistical analyses throughout this manuscript unless otherwise specified. Wilcoxon signed-rank test or Student’s t-test was used to determine the significance between the samples. *p*-values, false discovery rate (FDR), and fold change (FC) were calculated in analyses. Significant differences for all quantitative data were considered when *p < 0.05, **p < 0.01, ***p < 0.001, and ****p < 0.0001. The New WashU Epigenome Browser (http://epigenomegateway.wustl.edu) was used to extract and visualize representative sequencing tracks.

## Results

### Characterization of Critical Transcripts Associated With 5-Fluorouracil Resistance

In order to investigate potential RNA transcripts, such as protein-coding mRNA, long non-coding RNAs (lncRNAs), and small non-coding microRNAs (miRNAs) associated with the resistance to 5-Fluorouracil (5-FU) in colon cancer cells, we performed the long-insert strand-specific transcriptome sequencing and small non-coding RNA sequencing (smRNA-seq) with two biological replicates for the parental and 5-FU-resistant HCT15 (HCT15-FR) cells (Supplementary Figures S1A–D).

**FIGURE 1:**
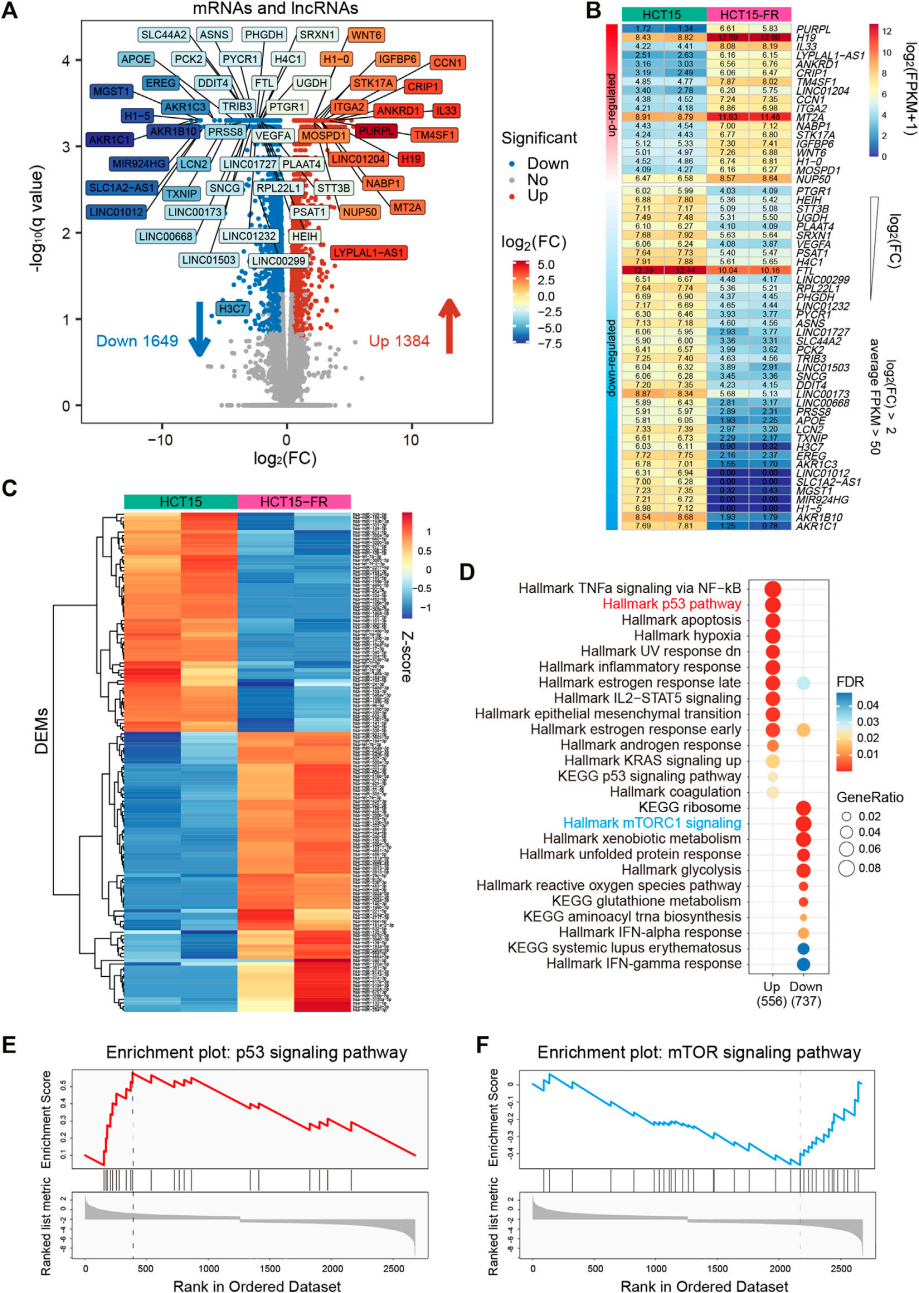
Identification and functional analysis of differentially expressed transcripts associated with 5-FU resistance in HCT15 cells. **(A)** Volcano plot representing the expression levels of all expressed mRNAs and lncRNA between the parental and 5-FU-resistant HCT15 cells. 1,649 downregulated mRNAs and lncRNAs are shown as blue dots, whereas 1,384 upregulated mRNAs and lncRNAs are shown as red dots. Filtered by absolute log_2_(FC) > 2, *p*-value < 0.05, and average FPKM >50, 57 significantly down- or upregulated mRNAs and lncRNAs are labeled in different colors based on log_2_ fold change (FC). **(B)** Heatmap of 57 critical mRNAs and lncRNAs was labeled in the volcano plot **(A)**, which are listed in a descending order based on log2(FC). The numeric values were log2(FPKM+1). **(C)** Heatmap showing the expression levels of 142 differentially expressed miRNAs (DEMs) in HCT15-FR cells compared to the parental cells. The expression values were normalized per row (Z-score). **(D)** Gene set enrichment analysis (GSEA) of HCT15-FR versus HCT15 cells was performed on DEG lists ranked by the log_2_(FC) using the R package “clusterProfiler” with HALLMARK and KEGG gene sets downloaded from MSigDB. Gene ratio refers to the ratio of the number of genes enriched in each term to the total number of genes in the term. **(E and F)** Representative GSEA plots upregulated p53 **(E)** and downregulated mTOR **(E)** signaling pathways using “WebGestalt” for DEGs ranked by the log_2_(FC).

A total of 3,033 differential expression mRNAs (DEGs) and lncRNAs (DELs) were identified simultaneously, among which 1,649 mRNAs and lncRNAs were downregulated and 1,384 mRNAs and lncRNAs were upregulated (Figure 1A, Supplementary Figures S1E, F and Supplementary Tables S1, S2). Filtered by absolute log_2_ (fold change) > 2, *p*-value < 0.05, average FPKM >50 in at least one group, we obtained 57 DEGs and DELs that might play critical roles in HCT15-FR cells (Figures 1A,B), including dramatically upregulated *IL33* (interleukin 33), *WNT6* (Wnt family member 6), *H1-0* (H1.0 linker histone), and the long non-coding RNA *PURPL* (p53 upregulated regulator of p53 levels), *H19* (H19 imprinted maternally expressed transcript) and *LINC01204*. Meanwhile, *AKR1B10* (aldo-keto reductase family 1 member B10), *H1-5* (H1.5 linker histone, cluster member), *FTL* (ferritin light chain), and the long non-coding RNA 1012 (*LINC01012*), SLC1A2 antisense RNA 1 (*SLC1A2-AS1*), *LINC00173* were significantly downregulated. Moreover, using smRNA-seq, we obtained 142 differentially expressed miRNAs (DEMs) in HCT15-FR versus HCT15 cells, such as hsa-miR-17-5p and hsa-miR-125b-5p (Figure 1C, Supplementary Figures S1G–H and Supplementary Tables S3, S4).

Gene set enrichment analysis (GSEA) using “clusterProfiler” (Yu et al., 2012) and WebGestalt (Wang et al., 2013) revealed that the upregulated DEGs in HCT15-FR cells were mainly enriched for gene sets associated with TNFa signaling *via* the NF-kB, signaling pathways of p53, IL2-STAT5, and KRAS, while those of the downregulated DEGs were associated with gene sets linked to mTORC1 signaling, xenobiotic, and glutathione metabolism (Figures 1D–F, Supplementary Figure S2C). We also performed GO analysis to elucidate the biological processes, which indicated that the regulation of secretion, programmed cell death, and epithelial to mesenchymal transition was upregulated, while the protein targeting the membrane, peptide, and the biosynthetic process was downregulated in HCT15-FR cells (Supplementary Figures S2A, B).

### Potential Chromatin Modifiers and Competitive Endogenous RNA

**FIGURE 2:**
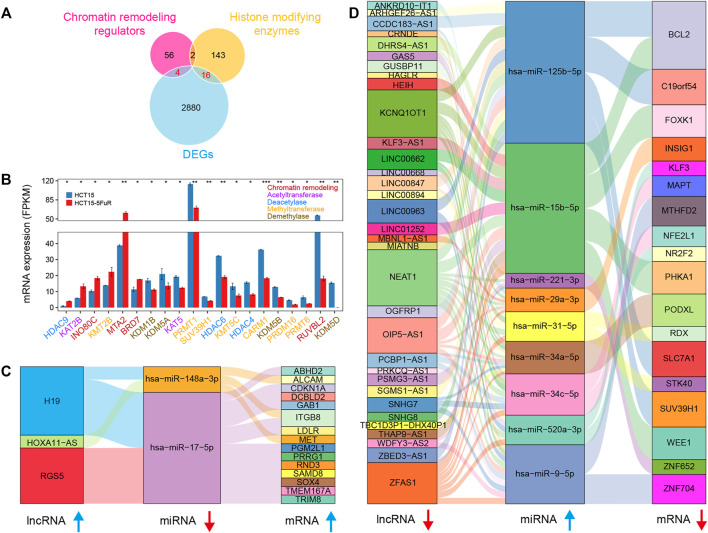
Differentially expressed epigenetic regulators and competing endogenous RNA (ceRNA). **(A)** Venn diagram illustrating the overlap among the known histone-modifying enzymes and chromatin remodeling regulators and DEGs. **(B)** Bar plot showing average expression levels (FPKM) of histone-modifying enzymes chromatin remodeling regulators identified in **(A)**, including histone acetyltransferase (purple), methyltransferase (orange), demethylase (brown), deacetylase (blue), and chromatin remodeling regulators (red). **(C and D)** Sankey diagram for the ceRNA network in HCT15-FR cells. Each rectangle represents an lncRNA, miRNA, or mRNA, and the connection degree of each RNA was displayed based on the size of the rectangle.

Based on the transcriptome sequencing data, we obtained 20 differentially expressed known chromatin-remodeling complexes and histone-modifying enzymes (Clapier et al., 2017; Stillman 2018), which might involve in the alteration of chromatin structure, epigenetic control of critical cellular functions, and the modification of histone and non-histone substrates (Figure 2A). Several chromatin-remodeling factors such as INO80C, MTA2, and BRD7 were significantly upregulated, while most of the differentially expressed histone-modifying enzymes were downregulated in 5-FU-resistant cells compared to the parental HCT15 cells (Figure 2B).

lncRNA has recently drawn increasing attention because it can sponge the miRNA pool and function as a ceRNA to communicate with the mRNA (Salmena et al., 2011; Wang et al., 2017). To better understand the effect of lncRNAs on mRNAs mediated by combination with miRNAs in HCT15-FR cells, we constructed the ceRNA network of lncRNAs–miRNAs–mRNA based on our sequencing data and the LncSEA (Chen et al., 2021) and miRWalk 2.0 (Dweep et al., 2011) databases (Figures 2C,D). The results indicated that the upregulated lncRNA H19 might promote 5-FU resistance by targeting the hsa-miR-148a-3p/MET axis (Figure 2C), whereas the downregulation of *BCL2* and *FOXK1* probably related to the hsa-miR-15b-5p upregulation, which could be targeted by lncRNA NEAT1 and/or LINC00662 (Figure 2D).

### Genome-Wide Alterations of Chromatin Accessibility

The ATAC-seq is currently the most popular technique for mapping genome-wide chromatin accessibility and provides information on nucleosome localization and transcription factor binding sites (TFBSs) on the chromatin, which relies on the fast and accurate quantification of insertion events of the hyperactive Tn5 transposase (Buenrostro et al., 2013; Wu et al., 2016; Li et al., 2017). To generate ATAC-seq libraries, we employed the parental and 5-FU-resistant HCT15 CCCs with two independent biological replicates for each group, and approximately 50,000 accessible chromatin regions (peaks) were identified for each sample (Supplementary Figure S5).

**FIGURE 3:**
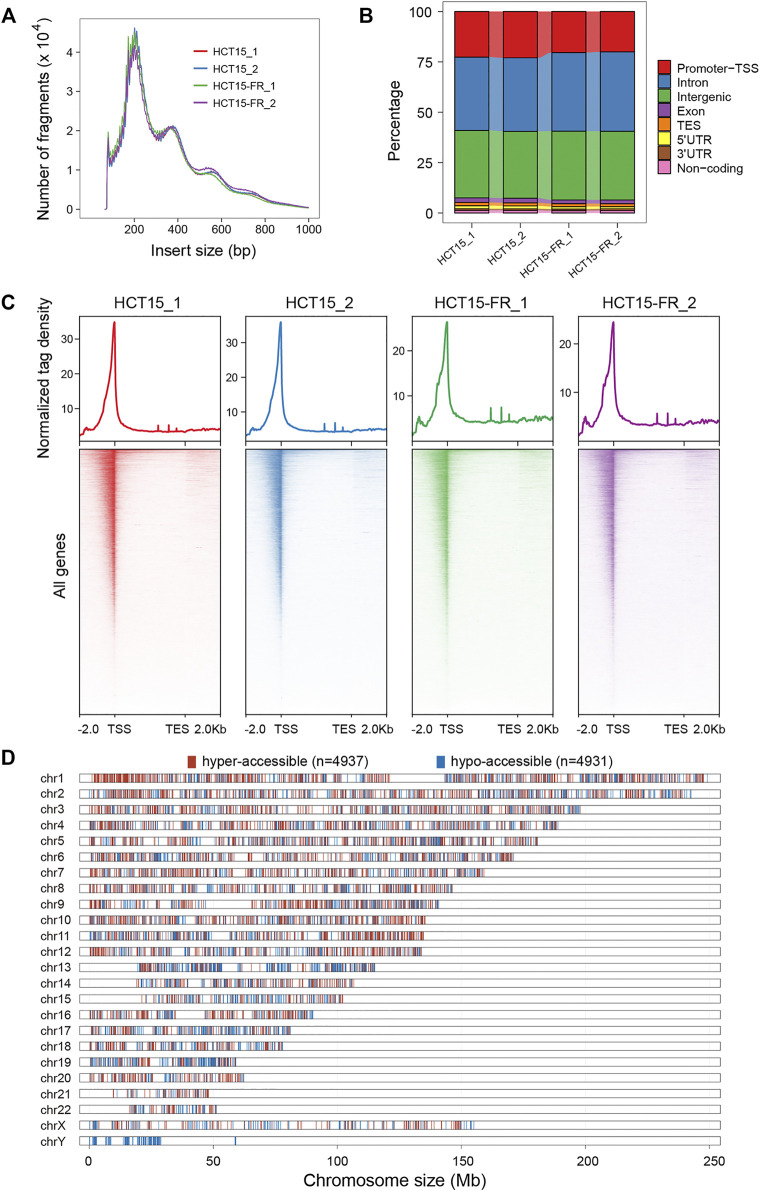
Chromatin accessibility landscape of HCT15 and HCT15-FR cells. **(A)** Fragment size distribution of ATAC-seq libraries obtained from each sample. Clear modulation of signals indicates mono-, di-, and tri-nucleosomes. **(B)** Proportion of ATAC-seq peaks to genomic features: promoters-TSS, TES, exon, intron, intergenic, 3′ UTR, 5′ UTR, and non-coding regions. TES, transcription end site. Promoter-TSS, peak summit located within upstream -1 Kb to +100 bp downstream of the transcription start site (TSS). **(C)** The normalized read density (top) and heatmaps (bottom) showing ATAC-seq signal across a genomic window within a 4-Kb region flanking the TES (from -2 Kb to +2 Kb). **(D)** Distribution of differentially accessible regions (DARs) over chromosomes in HCT15-FR cells compared to HCT15. The red and blue colors refer to hyper- and hypo-accessible regions, respectively.

The expected nucleosome banding patterns were examined by analysis of fragment size distribution, and the peaks with different lengths showed that the fragmented chromatin contained one or more nucleosomes, which were generated by the Tn5 transposase (Figure 3A). The similar distribution of fragment sizes indicates the reproducibility and reliability of our data in assessing chromatin accessibility. Genomic distribution analysis of ATAC-seq peaks over different types of functional elements suggested that the introns, intergenic, and promoter TSS regions were preferentially (more than 90%) accessible to Tn5 transposase (Figure 3B). Moreover, our results revealed that the majority of the accessible regions localize within a 2-Kb region flanking TSS (Figure 3C), which conformed to the pattern reported in previous studies in multiple organisms that chromatin accessibility by ATAC-seq is predictive of active transcription and most of the *cis*-regulatory elements are located in the vicinity of accessible promoter regions (Buenrostro et al., 2013; Doganli et al., 2017; Lu et al., 2017; Bozek et al., 2019).

**FIGURE 4:**
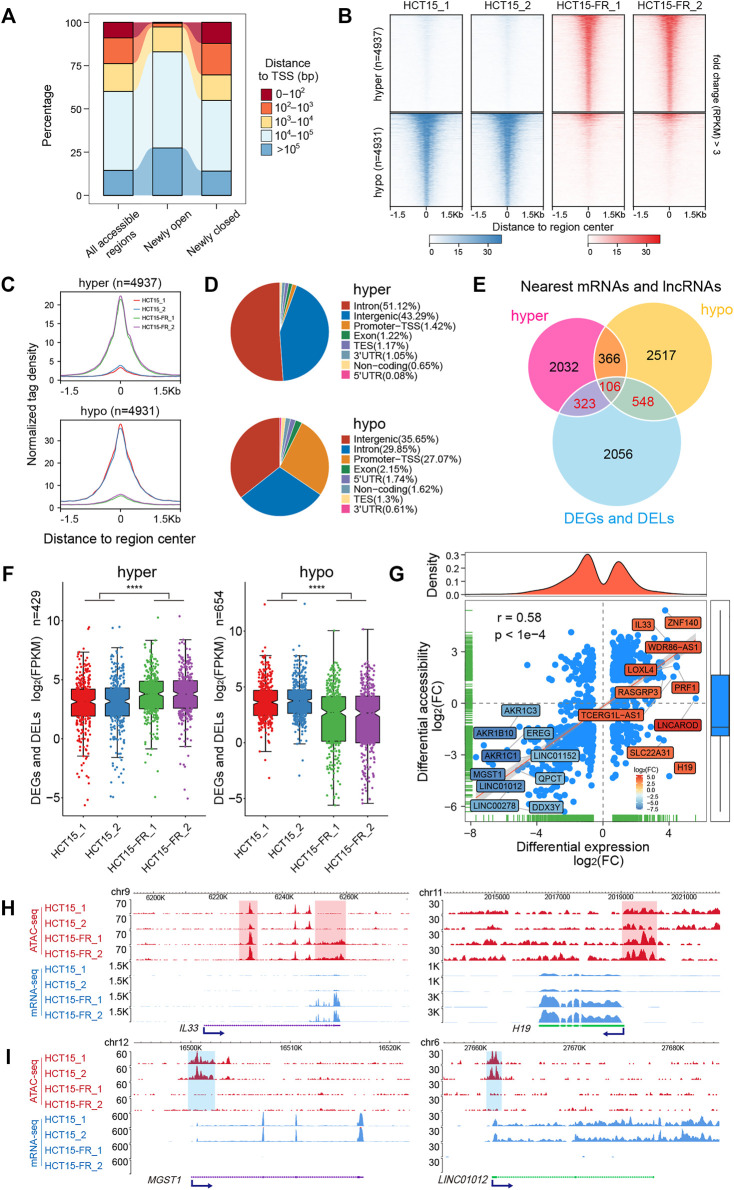
Genome-wide identification and analysis of differentially accessible regions (DARs). **(A)** Distance to nearest TSS of all accessible regions, differentially open, or closed regions. **(B)** Heatmaps indicating the normalized ATAC-seq signals (RPKM) in parental and 5-FU-resistant HCT15 cells over DARs. The top panel shows read signals over the 4,937 hyper-accessible regions, while the bottom panel shows read signals over the 4,931 hypo-accessible regions. Signals across a genomic window of ±1.5 Kb surrounding the center of DARs are shown in descending order. **(C)** Profiles of normalized tag density within a 3-Kb region flanking the hyper- (top) and hypo-accessible (bottom) regions. **(D)** Pie chart showing the annotation of hyper- (top) and hypo-accessible (bottom) sites within the indicated genomic regions: Promoters-TSS, TES, exon, intron, intergenic, 3′ UTR, 5′ UTR, and non-coding regions. TES, transcription end site. Promoter-TSS, peak summit located upstream -1 Kb to +100 bp downstream of the TSS. **(E)** Venn diagram illustrating the overlap among DEGs, DELs, differentially accessible regions nearest mRNAs, and lncRNAs. **(F)** Box plots for expression levels of DEGs and DELs relative to hyper- (left) and (right) hypo-accessible regions identified in **(E)**. *p*-values were calculated by Wilcoxon’s signed-rank test in R, ****p < 0.0001. **(G)** Correlation analysis between DARs and their nearest DEGs and DELs. Blue dots represent mRNAs or lncRNAs that are differentially expressed and associated with the changes in chromatin accessibility. The top- and bottom-ranked 15 DEGs or DELs are labeled and shown in different colors according to the log_2_(FC). Pearson’s correlation coefficient (r) and the corresponding *p*-value were calculated by R. (H and I) The New WashU Epigenome Browser tracks showing ATAC-seq (red) and RNA-seq (blue) signals of representative upregulated **(H)** *IL33* and *H19*, and downregulated **(I)** *MGST1* and *LINC01012*. Hyper- and hypo-accessible regions are shaded in red and blue, respectively. Blue arrows indicate the TSS and direction of transcription.

To address whether the alteration of chromatin accessibility was correlated with transcription changes genome-wide, we assessed differential accessibility (RPKM) between the parental and 5-FU-resistant HCT15 cells. In total, 4,937 regions showed increased chromatin accessibility > 3-fold in HCT15-FR cells, while 4,931 regions were > 3-fold more accessible in the parental cells; thus, we defined these chromatin states as “hyper-accessible” and “hypo-accessible”, respectively (Figure 3D, Figures 4A–C). Genomic annotation of these differentially accessible regions (DARs) demonstrated that largely the gene-distal and intragenic regions (gene bodies) exhibited differential accessibility with a relatively high proportion of hypo-accessible regions were located within promoter-proximal regions (Figures 4A,D and Supplementary Table S6), suggesting that DARs presumably represent distal regulatory elements such as enhancers, which were often located at great distances from the genes they regulate (Denny et al., 2016).

### The DARs Were Positively Correlated With Nearest DEGs and DELs

To further explore the potential relationship between chromatin accessibility alterations and differential gene expression, we assigned the DARs (located within 100 Kb of the TSS) to the nearest mRNAs and lncRNAs according to their genomic locations. A total of 429 and 654 nearby DEGs and DELs were identified to be associated with the hyper- and hypo-accessible regions, respectively (Figure 4E). Notably, the results showed that most of the DEGs and DELs associated with hyper-accessible regions were significantly (*p* < 0.0001) upregulated, while the DEGs and DELs associated with hypo-accessible regions displayed downregulated expression levels in all biological replicates (Figure 4F), indicating that mRNAs and lncRNAs close to the open chromatin regions tend to have higher expression levels, and vice versa. Upon further correlation analysis, it was confirmed that the DARs were positively (Pearson correlation coefficient *r* = 0.58, *p* < 0.0001) associated with their nearest DEGs and DELs (Figure 4G), suggesting that the alterations in chromatin accessibility contribute to the concomitant differential expression of the mRNAs and lncRNAs in HCT15-FR cells, such as *IL33*, *MGST1*, H19, and LINC01012 (Figures 4G–I).

### Signaling Pathways of DEGs Positively Associated With DARs

**FIGURE 5:**
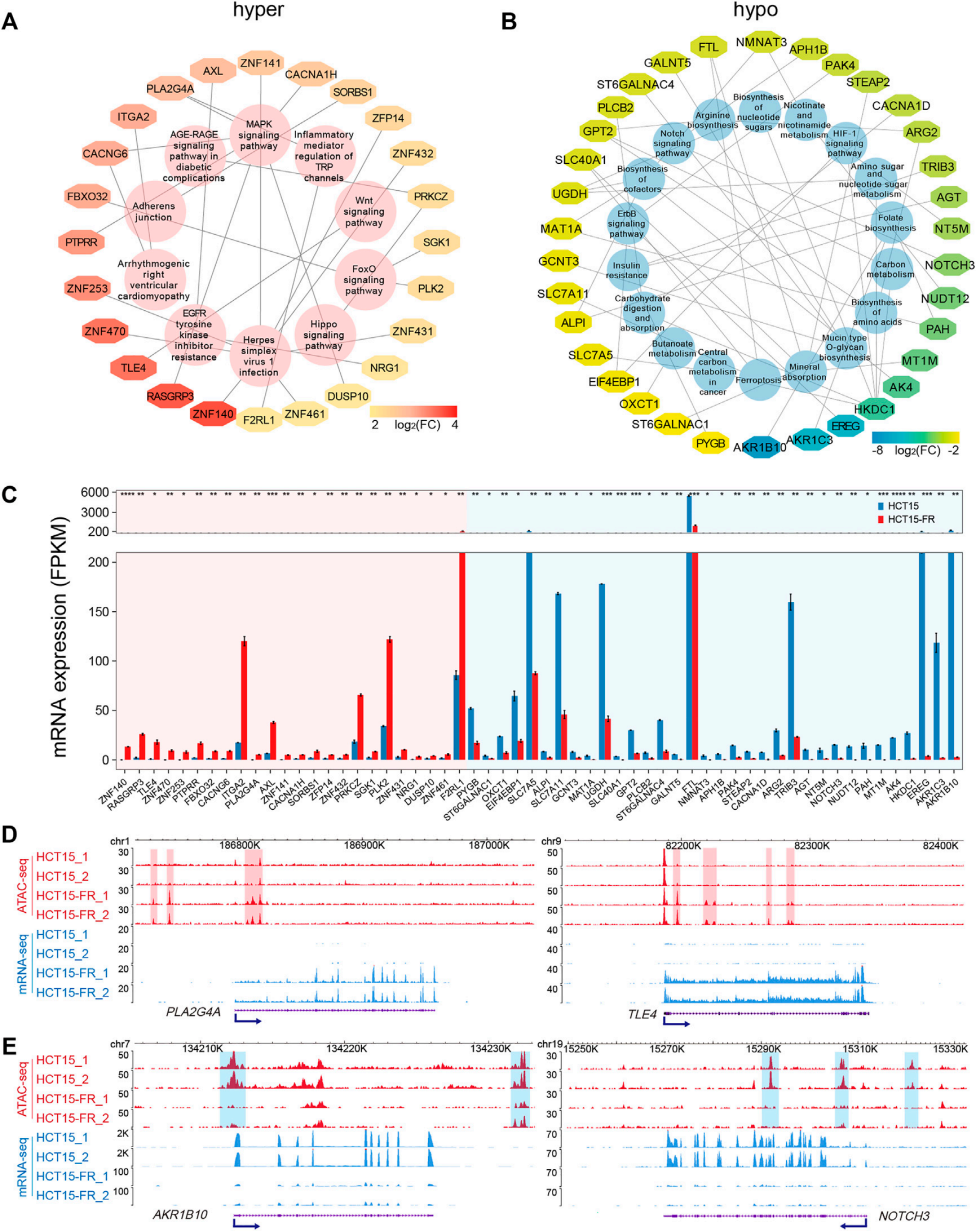
Key KEGG pathways associated DEGs related to DARs. **(A,B)** KEGG pathways associated DEGs related to hyper- (A) and hypo-accessible **(B)** regions. DEGs are shown in different colors based on log_2_(FC) of average FPKM. **(C)** Bar plot showing average expression levels of DEGs that enriched in KEGG pathways **(A,B)** and related to hyper- (red shade) and hypo-accessible (blue shade) regions. All DEGs are listed in descending order according to log_2_(FC). **(D,E)** Genomic snapshots of ATAC-seq (red) and RNA-seq (blue) signals of representative upregulated **(D)** *PLA2G4A* and *TLE4*, and downregulated **(E)** *AKR1B10* and *NOTCH3*. Hyper- and hypo-accessible regions are shaded in red and blue, respectively. Blue arrows indicate the TSS and direction of transcription.

To examine signal pathways of the DEGs correlated to DARs in HCT15-FR cells, we performed KEGG pathway enrichment analysis for up- and downregulated DEGs associated with hyper- and hypo-accessible regions, respectively. The results showed that the hyper-accessible regions associated upregulated DEGs were significantly (*p* < 0.05) enriched in EGFR tyrosine kinase inhibitor resistance, signaling pathways of MAPK, FOX, and WNT (Figure 5A), while the hypo-accessible regions associated downregulated DEGs mainly involved in insulin resistance, biosynthesis of amino acids, nucleotide sugars and cofactors, and the signaling pathways of Notch, HIF-1, and ErbB (Figure 5B). Integrative ATAC-seq and RNA-seq analysis revealed that a total of 58 key DEGs were enriched in the KEGG pathways with the absolute value of log_2_(FC) > 2 (Figures 5A,B). Of these, 34 downregulated and 24 upregulated DEGs were positively associated with hypo- and hyper-accessible regions, respectively, including *TLE4*, *ITGA2*, *PLK2*, *SLC7A5*, *FTL*, and *NOTCH* (Figures 5C–E, Supplementary Figure S3). This analysis showed global alterations of chromatin accessibility impacted gene expression of many components of signaling cascades, which played a critical role in acquired resistance to 5-FU in HCT15 cancer cells.

### Transcription Factor Families Associated With 5-FU-Induced Chromatin Accessibility Changes

**FIGURE 6:**
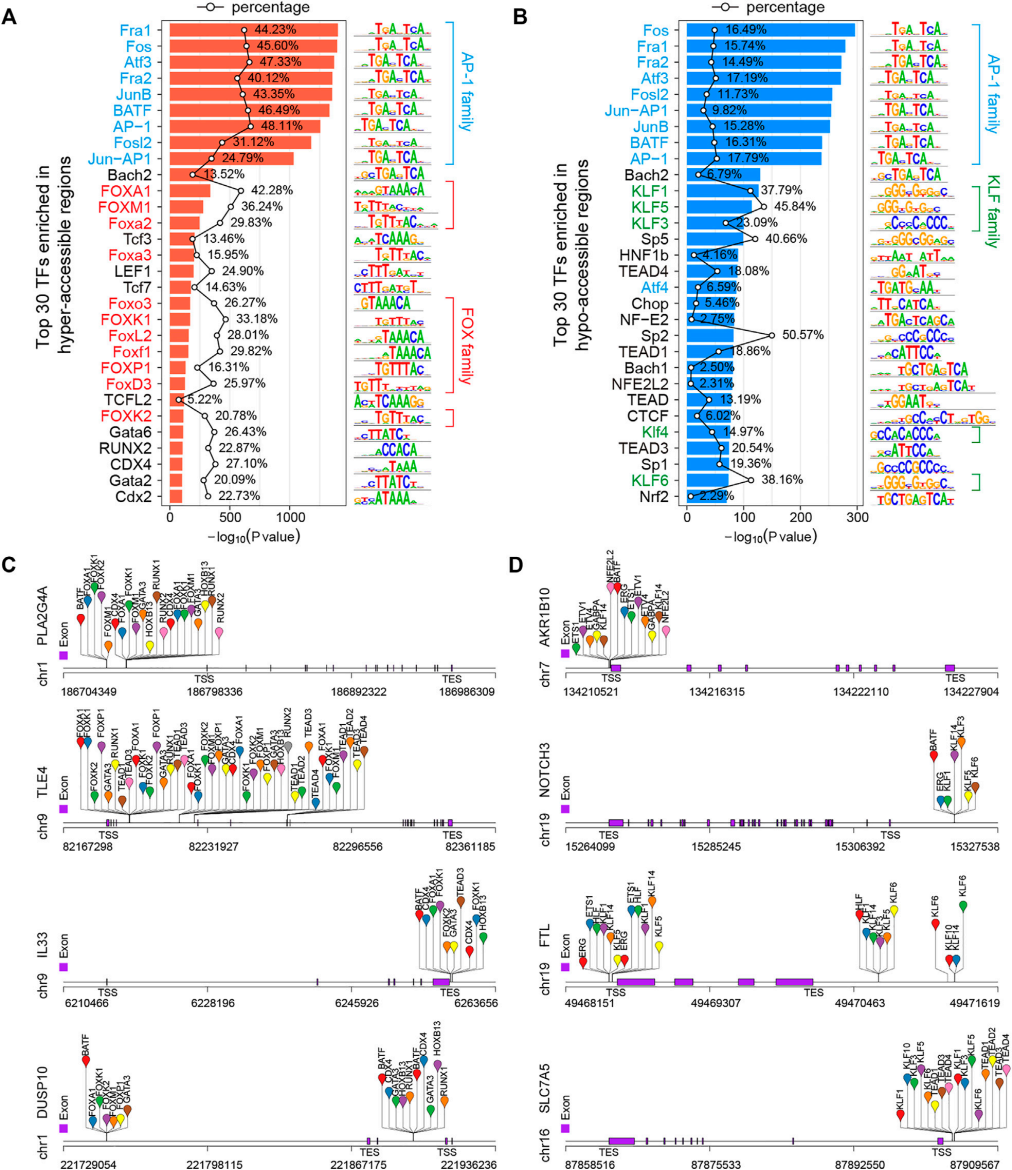
Transcription factor (TF) motif enrichment analysis of DARs in 5-FU-resistant HCT15 cells. **(A,B)** Top 30 enriched known TF motifs of hyper- (A) and hypo-accessible **(B)** regions. The AP-1, FOX, and KLF family members are shaded blue, red, and green, respectively. The polygonal chain shows the percentages of target sequences with motif, and the *p*-value was calculated using the cumulative binomial distribution in HOMER v4.10. (C and D) Predicted transcription factor binding sites (TFBSs) of representative up- **(C)** and downregulated **(D)** DEGs that positively correlated to chromatin accessibility changes.

A TF is a protein that recognizes a specific sequence, such as a motif, in the promoter or enhancer of a target gene and is able to promote or repress its transcriptional activity (Tsankov et al., 2015; Whyte et al., 2013). Chromatin accessibility to DNA is a prerequisite for TF binding, and nucleosome occupancy of DNA is thought to hinder TF binding and transcription, so the regulation of chromatin accessibility is key to gene regulatory mechanisms (Chen et al., 2018; Huertas et al., 2020). The integration of open chromatin regions identified by ATAC-seq and TF binding motifs can predict TF occupancy and TFBSs on the chromatin (Bao et al., 2015; Corces et al., 2018; Qu et al., 2017). To identify potential TFs responsible for observed changes in gene expression and chromatin accessibility, we performed DNA motif enrichment analysis to find consensus TF binding motifs that were enriched in the DARs using HOMER v4.10 (Heinz et al., 2010) over a 200-bp region surrounding the ATAC-seq peak summits. Finally, 268 and 272 significantly (*p* < 0.01) enriched TF motifs were identified in hyper- and hypo-accessible regions, respectively (Supplementary Table S7). As expected, the activator protein-1 (AP-1) family members, such as Fos (c-Fos, FosB, FosB2, Fra1, and Fra2), ATF (ATFa, ATF2, ATF3, and BATF) and Jun (c-Jun, JunB, and JunD), were significantly enriched in both hyper- and hypo-accessible regions (Figures 6A,B), which selectively recognize and bind to the target sequence 5′-TGACTCA-3′ and play a critical role in a wide array of physiological and pathophysiological processes (Eferl and Wagner 2003; Lambert et al., 2018; Papoudou-Bai et al., 2017). In addition, the hyper-accessible regions were also enriched with forkhead box (FOX) family members, including FOXA1, FOXM1, and FOXP1 (Figure 6A), while the hypo-accessible regions were enriched for Krüppel-like factor (KLF) family members, such as KLF1, KLF3, and KLF3 (Figure 6B). Furthermore, in order to obtain potential regulators of the DEGs associated with DARs, we performed the analysis of the binding sites of top 50 TFs (Supplementary Table S7) using HOMER (Heinz et al., 2010). The results showed that the hyper-accessible sites within TSS ±3-kb regions of key upregulated DEGs such as *PLA2G4A*, *TLE4*, and *IL33* were significantly enriched by TFBSs of FOX family members, RUNX2, and/or GATA3 (Figures 5D, 6C). Meanwhile, the KLF and TEAD family members were potentially bound to the hypo-accessible sites associated with downregulated DEGs, such as *AKR1B10*, *NOTCH3*, and *SLC7A5* (Figures 5E, 6D).

### Differentially Expressed Transcription Factors and Their Target DEGs

**FIGURE 7:**
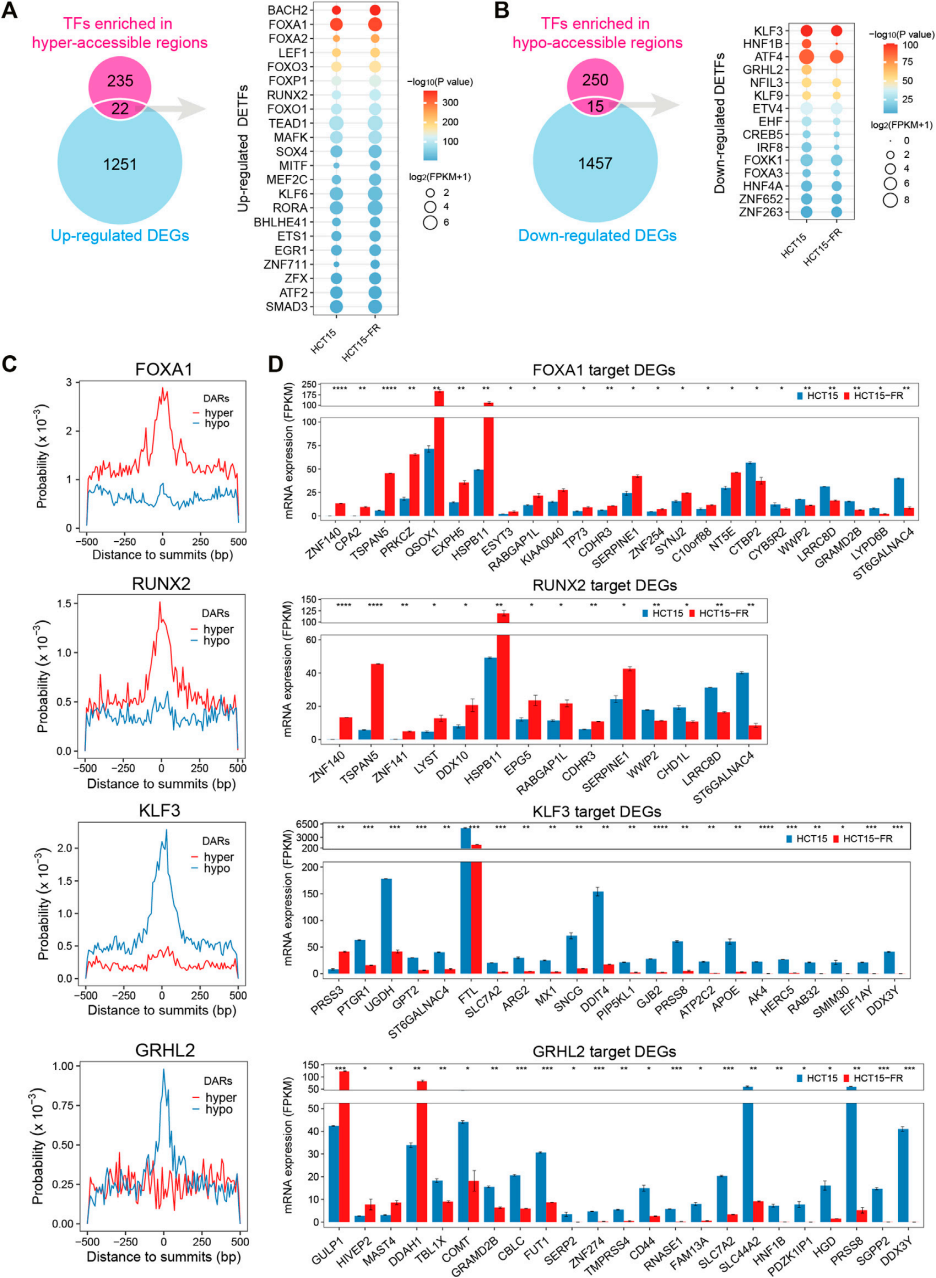
Identification of differentially expressed transcription factors (DETFs) and their target DEGs. **(A)** Venn diagram showing overlap of the corresponding TFs for each motif in hyper-accessible regions and upregulated DEGs. The dot plot indicates the identified upregulated DETFs. The size of each dot represents the mRNA expression level of the enriched motif cognate TFs. Only TFs motif enrichment *p*-value < 0.05 was included. The color of each dot represents different *p*-values for enriched motifs. **(B)** Venn diagram showing overlap of the corresponding TFs for each motif in hypo-accessible regions and downregulated DEGs. The dot plot indicates the identified downregulated DETFs. **(C)** Distribution probability of FOXA1, RUNX2, KLF3, and GRHL2 binding motifs surrounding ATAC-seq peak summits (±500 is shown) in DARs. **(D)** Bar charts showing the expression levels of potential TF (FOXA1, RUNX2, KLF3, and GRHL2) target DEGs in DARs predicted by HOMER. Only DEGs with the distance of TFBSs to TSS less than 3 Kb were included. All DEGs are displayed in a descending order based on log_2_(FC).

To obtain the DETFs that might be connected with the alteration of chromatin accessibility and gene transcription, the identified motif cognate TFs (*p* < 0.05, Supplementary Table S7) were intersected with the DEGs (Supplementary Table S2). The results showed 22 upregulated (Figure 7A, Supplementary Figure S5A) and 15 downregulated (Figure 7B, Supplementary Figure S5B) TFs were associated with the hyper- and hypo-accessible regions, respectively. For instance, the upregulated FOX family members (such as FOXA1, FOXO3, and FOXP1) and RUNX2 were significantly enriched in hyper-accessible regions, while the downregulation of KLF3, HNF1B, and GRHL2 might be responsible for the decreases in chromatin accessibility. As expected, more in-depth analysis revealed that the FOX family members and RUNX2 were generally distributed more frequently around ATAC-seq peak summits in hyper-accessible regions, while the KLF family members and GRHL2 showed higher binding probability in hypo-accessible regions (Figure 7C, Supplementary Figure S4A). TF footprinting analysis using HINT (Gusmao et al., 2016) also revealed that the members of FOX and KLF families such as FOXA1, FOXO3, KLF1, and KLF3 were significantly enriched in hyper- and hypo-accessible regions, respectively. By comparing the footprint average profiles, we found that there were a higher number of normalized ATAC-seq counts for particular upregulated TFs identified by HOMER, including FOXA2, RUNX2, and BACH2 (Figure 7A, Supplementary Figure S6A), within the hyper-accessible chromatin regions, indicating higher activity of these TFs in HCT15-FR cells. On the contrary, some downregulated TFs such as KLF3, HNF1B, and ETV4 had lower ATAC-seq signals within the hypo-accessible regions (Figure 7B, Supplementary Figure S6B). Moreover, a majority of predicted target genes of FOXA1 and RUNX2 were significantly upregulated, including *PRKCZ*, *HSPB11*, and *CDHR3*, whereas most of KLF3 and GRHL2 targets were downregulated, such as *FTL*, *SLC44A2*, and *HNF1B* (Figure 7D), suggesting that the differential expression patterns of TFs and their target DEGs were closely correlated with the alterations of chromatin accessibility in HCT15-FR cells (Figure 6D).

### Protein–Protein Interaction and Co-Expression Analysis of DETFs Related to DARs and 5-FU Resistance

**FIGURE 8:**
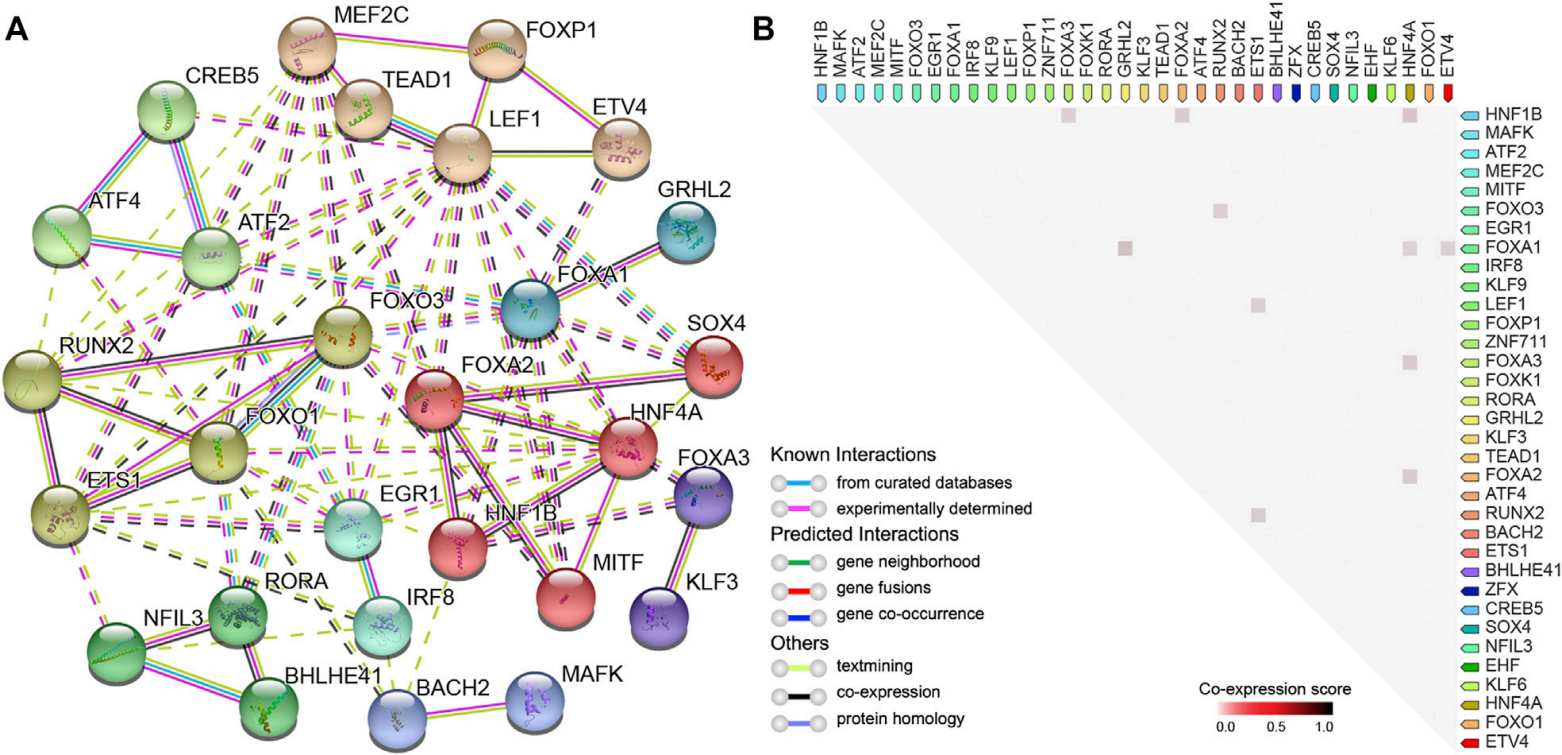
Protein–protein interaction (PPI) network and correlation analysis of DETFs. **(A)** PPI network representing DETFs (in Figures 7A,B) identified by HOMER enriched in DARs. In the network, the nodes indicate proteins, and different colored edges represent protein-protein associations among neighboring nodes. Cluster analysis using the Markov clustering (MCL) Algorithm with default inflation parameters. The dotted line represents edges between clusters. **(B)** Observed co-expression in *Homo sapiens* identified by STRING for DETFs associated with DARs. The co-expression scores between proteins were calculated based on gene expression patterns, and protein co-regulation was derived from ProteomeHD.

In order to reveal the PPI network and co-expression of the identified DETFs (*p* < 0.01), we made use of the STRING database (Szklarczyk et al., 2017), which is widely used to analyze known and predicted physical interactions and functional associations between proteins. The network indicated that clustered and upregulated RUNX2/FOXO3/ETS1 potentially cooperated to regulate gene expression in hyper-accessible regions, while the loss-of-function of HNF1B/HNF4A probably contributed to the downregulation of key DEGs in HCT15-FR cells (Figures 7A,B, 8A). Moreover, relatively high co-expression scores were also observed for RUNX2/ETS1, RUNX2/FOXO3, HNF1B/HNF4A, and HNF1B/FOXA3 (Figure 8B), suggesting that the DETFs associated with DARs might work together and be responsible for 5-FU resistance in colon cancer cells.

## Discussion

Although numerous molecular mechanisms involved in the process of 5-FU resistance in cancer have been reported (Yoo et al., 2004; De Angelis et al., 2006; Chen et al., 2019; Blondy et al., 2020; Vodenkova et al., 2020; Zhu et al., 2020), the development of resistance to 5-FU is almost inevitable in advanced colon cancer patients, which remains an unresolved issue. In recent years, a growing number of studies have demonstrated that the chromatin accessibility changes play a critical role in acquired drug resistance in cancer (Jing et al., 2018; Zhang L. L et al., 2020; Gallon et al., 2021), yet we still lack precise knowledge of how the accessible chromatin state can be integrated with transcriptional regulatory networks into coherent gene expression programs in 5-FU-resistant colon cancer cells.

Compared with other techniques, such as DNase-seq and FAIRE-seq, ATAC-seq is the most widely used method for determining chromatin accessibility across the genome due to its high accuracy, fast speed, and low input cell number requirement (Buenrostro et al., 2013). In the present study, we integrated chromatin accessibility and transcriptomics *via* ATAC-seq in combination with RNA-seq to determine whether the changes in chromatin accessibility correlated with differential gene expression and revealed candidate TF drivers between the parental and 5-FU-resistant HCT15 cells.

Based on the analysis of differential expression levels of mRNAs, lncRNAs, and miRNAs, we identified a number of dramatically upregulated transcripts associated with 5-FU resistance in HCT15 cells, such as *IL33*, H19, and hsa-miR-17-5p. Meanwhile, there were many significantly downregulated transcripts, including *FTL*, LINC01012, and hsa-miR-125b-5p. Moreover, we also identified some critical signaling pathways (such as p53 and mTOR), differentially expressed histone-modifying enzymes, and chromatin remodeling regulators, which might be involved in epigenetic regulatory processes in HCT15-FR cells.

The ceRNA hypothesis postulates that not only mRNAs but also other RNA transcripts, such as lncRNAs and circRNA, can hinder miRNA function by virtue of shared microRNA response elements as natural miRNA sponges (Salmena et al., 2011; Hansen et al., 2013; Tay et al., 2014). To better explain the interaction between lncRNAs, mRNAs, and miRNAs, we established an lncRNA–miRNA–mRNA ceRNA network mediated by up- and downregulated lncRNAs at the transcriptome-wide level, which lay a useful foundation for understanding the regulatory function of ceRNA associated with 5-FU resistance in HCT15 cells. For instance, as one of the earliest discovered lncRNAs, H19 is overexpressed in many cancers and has been recognized as a multifunctional regulator (Wang et al., 2016; Liu et al., 2021). Our ceRNA network indicated that H19 potentially regulated the mRNA expression of MET, SOX4, and TRIM8 by miR-148a-3p and/or miR-17-5p in a ceRNA manner. Similarly, upregulation of hsa-miR-125b-5p and hsa-miR-15b-5p could interact with the majority of DELs and DEGs, suggesting that they have a significant effect on the 5-FU resistance in HCT15 cells, but their individual contribution remains unclear.

Genome-wide profiling of chromatin accessibility showed that consistent with previous reports (Buenrostro et al., 2013; Jing et al., 2018), ATAC-seq signals mainly enriched in the promoter-TSS and distal regulatory elements, such as enhancers. Combined with transcriptome sequencing data, a strong positive correlation (r = 0.58, *p* < 0.0001) was found between DARs and their nearest differentially expressed transcripts (mRNAs and lncRNAs), suggesting that increased chromatin accessibility was accompanied by higher expression of mRNAs and/or lncRNAs. On the other hand, the closed chromatin restricts the binding of transcriptional regulators to the promoter and/or enhancer, which cause gene silencing such as LINC01012, *H1-5*, and *AKR1B10* in HCT15-FR cells. For smRNA-seq, we also performed correlation analysis using the Pearson correlation coefficient, but there was no statistically significant correlation between DARs and DEMs (data not shown). In addition, we screened some DEGs positively correlated with DARs and their functionally enriched signaling pathways.

By analyzing accessible chromatin regions, we can not only identify possible regulatory elements but also predict binding sites of TFs, which play a critical role in cancer drug resistance (Fan et al., 2017; Lambert et al., 2018; Huh et al., 2019; Bi et al., 2020). In this study, the motif discovery software HOMER was used for known motif enrichment analysis of the DARs to identify potential TFs and their target DEGs. Besides the AP-1 family, a common TF family in motif analysis of ATAC-seq, members of FOX and KLF families were significantly enriched in hyper- and hypo-accessible regions in HCT15-FR cells compared to parental HCT15 cells. In addition, we identified 21 up- and 13 downregulated TFs and their target DEGs related to the hyper- and hypo-accessible regions, respectively, including FOXA1, RUNX2, KLF3, and HNF1B, which we would like to pay more attention to. Furthermore, we constructed the PPI network of DETFs and analyzed their co-expression using the STRING database. The results showed that RUNX2/FOXO3/ETS1 and HNF1B/HNF4A exhibited strong correlations and relatively high levels of co-expression, suggesting that these DETFs played an important regulatory role in differential transcript expression, such as *PRKCZ*, *HSPB11*, *FTL*, and *SLC44A2*, associated with genome-wide chromatin accessibility changes.

According to previous reports and our data, we concluded that DARs could promote and/or suppress the expression of critical transcripts associated with 5-FU resistance by affecting the ability of TFs and transcriptional regulators to recognize and bind DNA in promoters and/or enhancers in 5-FU-resistant HCT15 cells. The current study provides the first description of the chromatin accessibility changes and differential transcript expression associated with 5-FU resistance in the HCT15 cells. However, much work remains to be done to fully understand the physiological and pathophysiological functions of ceRNAs, DARs, DETFs, and their targets in 5-FU-resistant HCT15 and other colon cancer cell lines.

To sum up, genome-wide chromatin accessibility profiling by ATAC-seq allows us to identify the accessible regulatory elements and TFs that are probably responsible for the spatial and temporal regulation of transcription, which provides a novel strategy to study transcriptome and accessible chromatin landscapes associated with cancer drug resistance. Our transcriptome sequencing and ATAC-seq data contribute to close the loop for the association between TF binding, chromatin, and transcriptional states, representing new insights into non-genetic mechanisms of acquired resistance to 5-FU in colon cancer cells.

## Conclusion

The integration of ATAC-seq and transcriptome sequencing represents a novel strategy to investigate the chromatin accessibility changes and different transcriptional regulations in cancer drug resistance. The application of these techniques allows us to identify TF-binding sites, *cis*-regulatory elements, ceRNAs, and therapeutic targets that are probably responsible for the acquired resistance to 5-FU in colon cancer cells. Our data provided clear insights and valuable resources for an improved understanding of the non-genetic mechanisms of 5-FU resistance and potential epigenetic targets for colon cancer therapy.

## Data Availability

All data supporting its findings in this study are available within the article and its supplementary files or from the corresponding author on reasonable request. All sequencing data have been deposited in the Gene Expression Omnibus (GEO) repository under the accession number GSE190951.
