# Supplementary figures and images for: Integrated Chromatin Accessibility and Transcriptome Landscapes of 5-Fluorouracil-Resistant Colon Cancer Cells
Source: Front Cell Dev Biol. 2022 Feb 17;10:838332. doi: 10.3389/fcell.2022.838332 (PMC8891516; doi:10.3389/fcell.2022.838332)

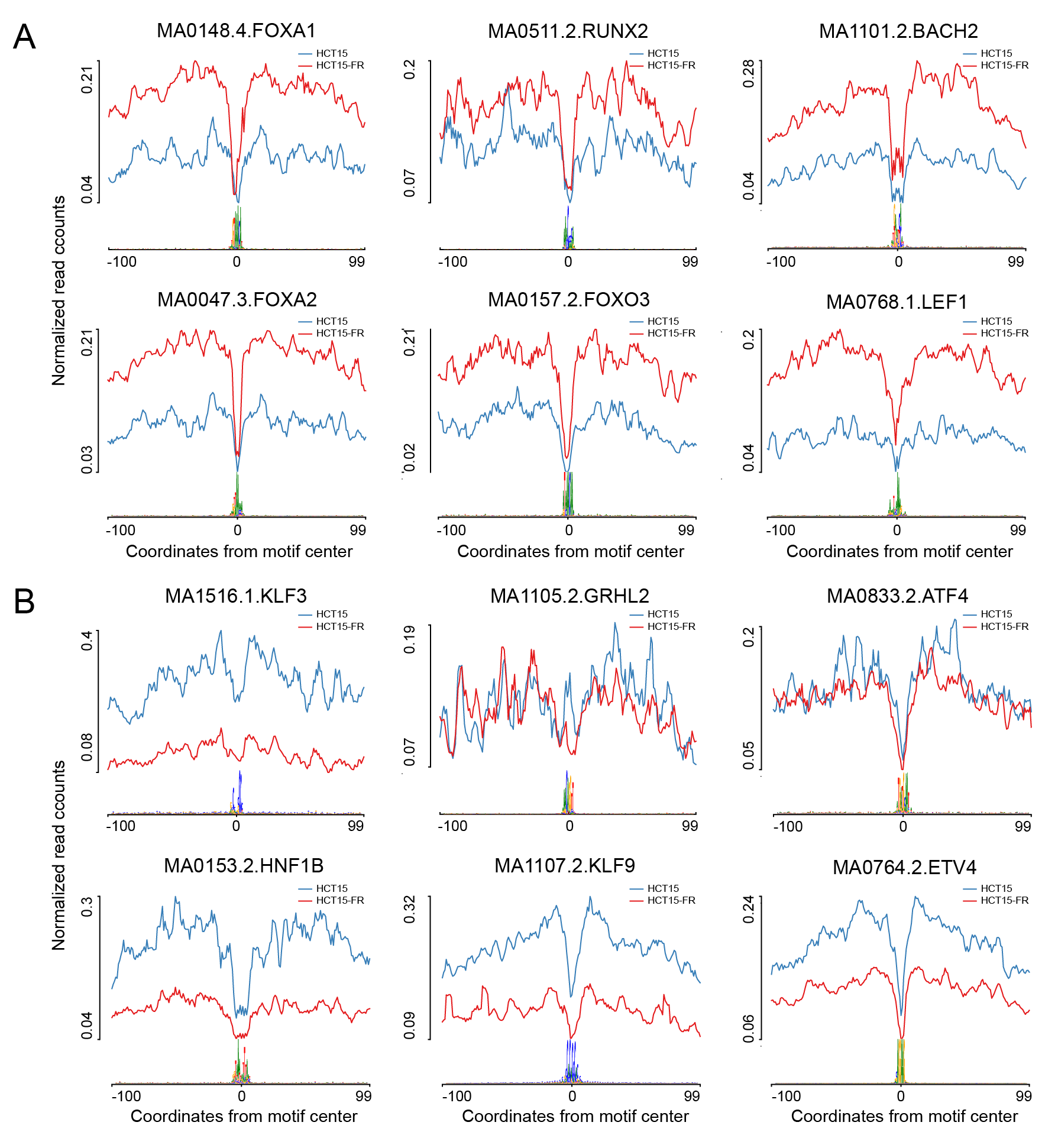

Supplement: Supplementary file 3 [file Image6.TIF]

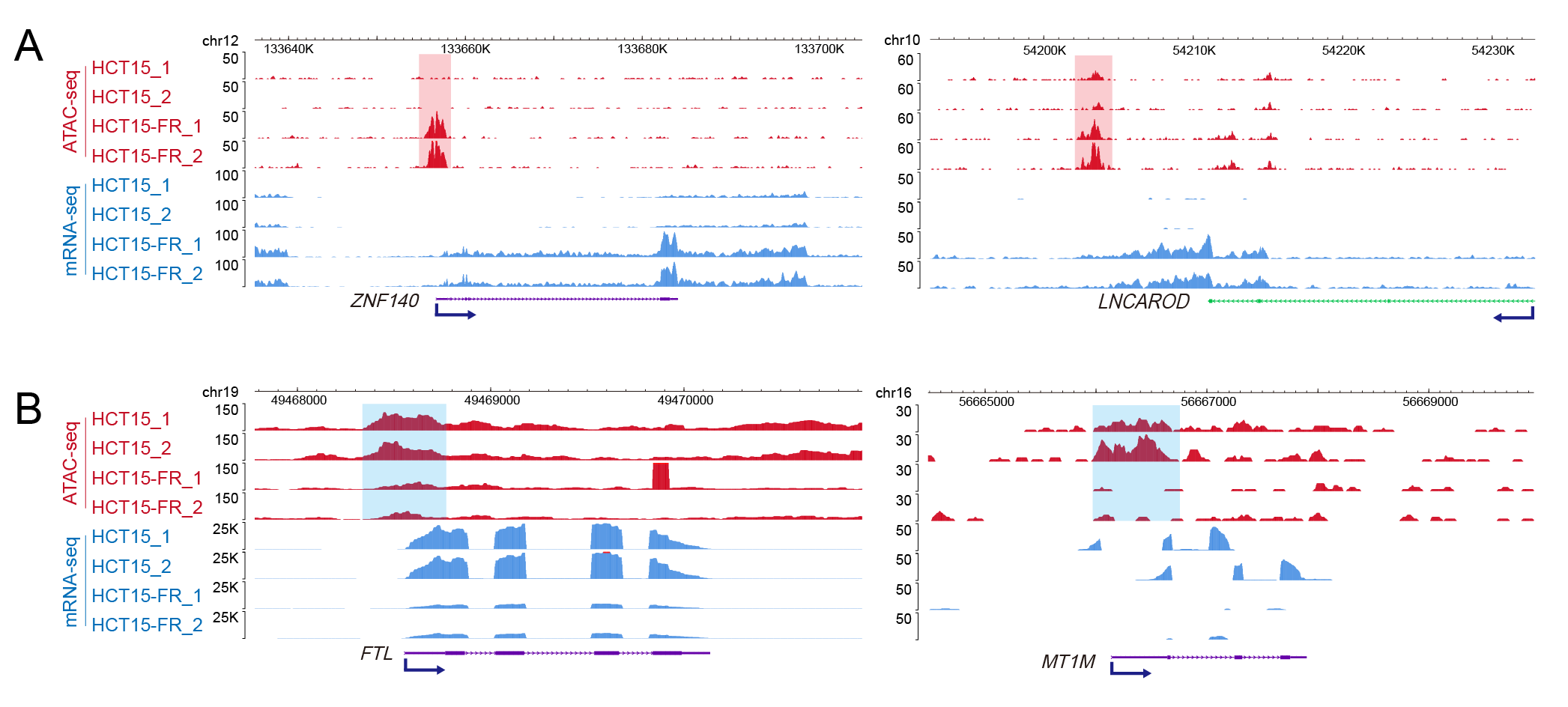

Supplement: Supplementary file 4 [file Image3.TIF]

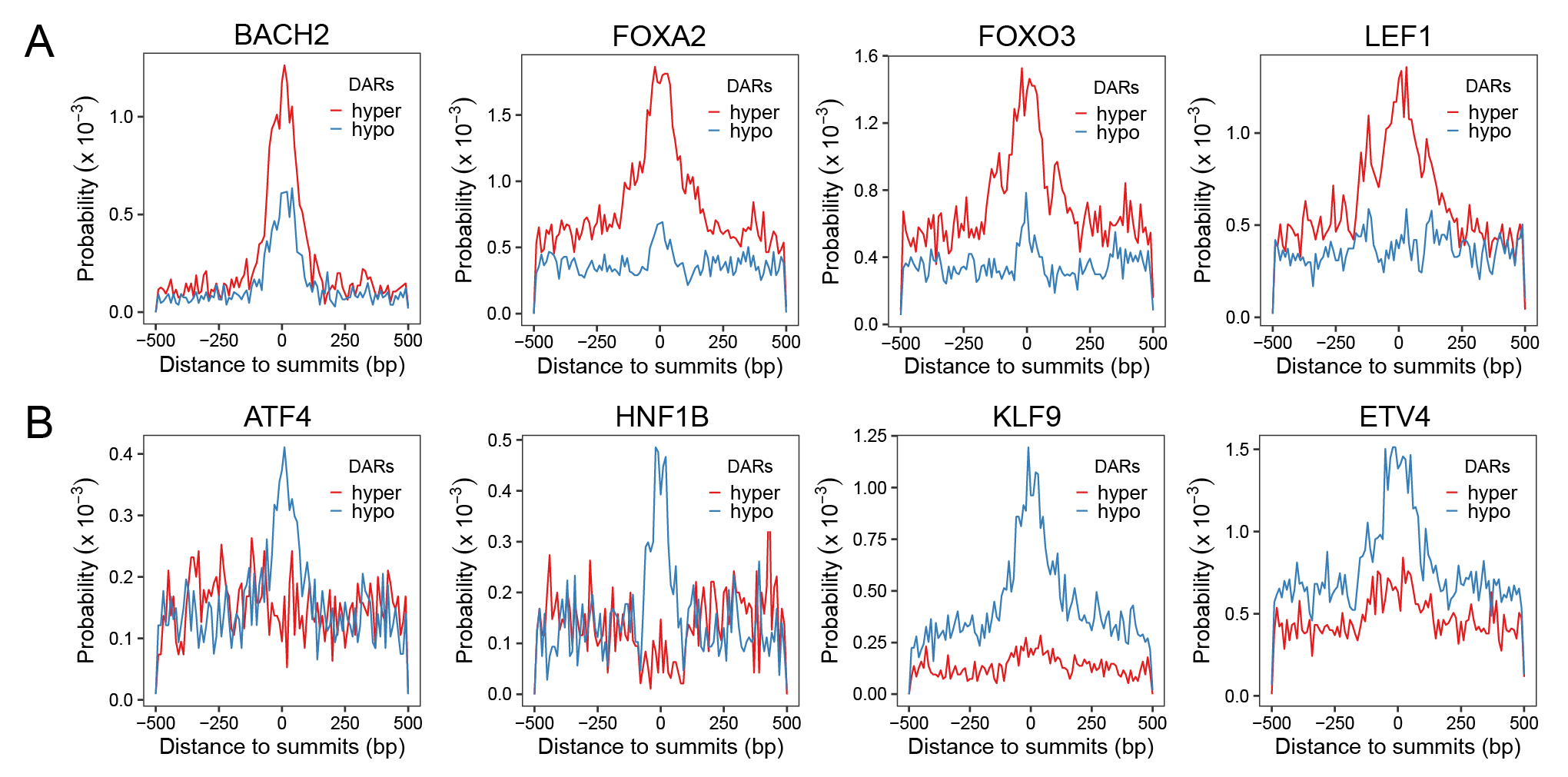

Supplement: Supplementary file 5 [file Image4.TIF]

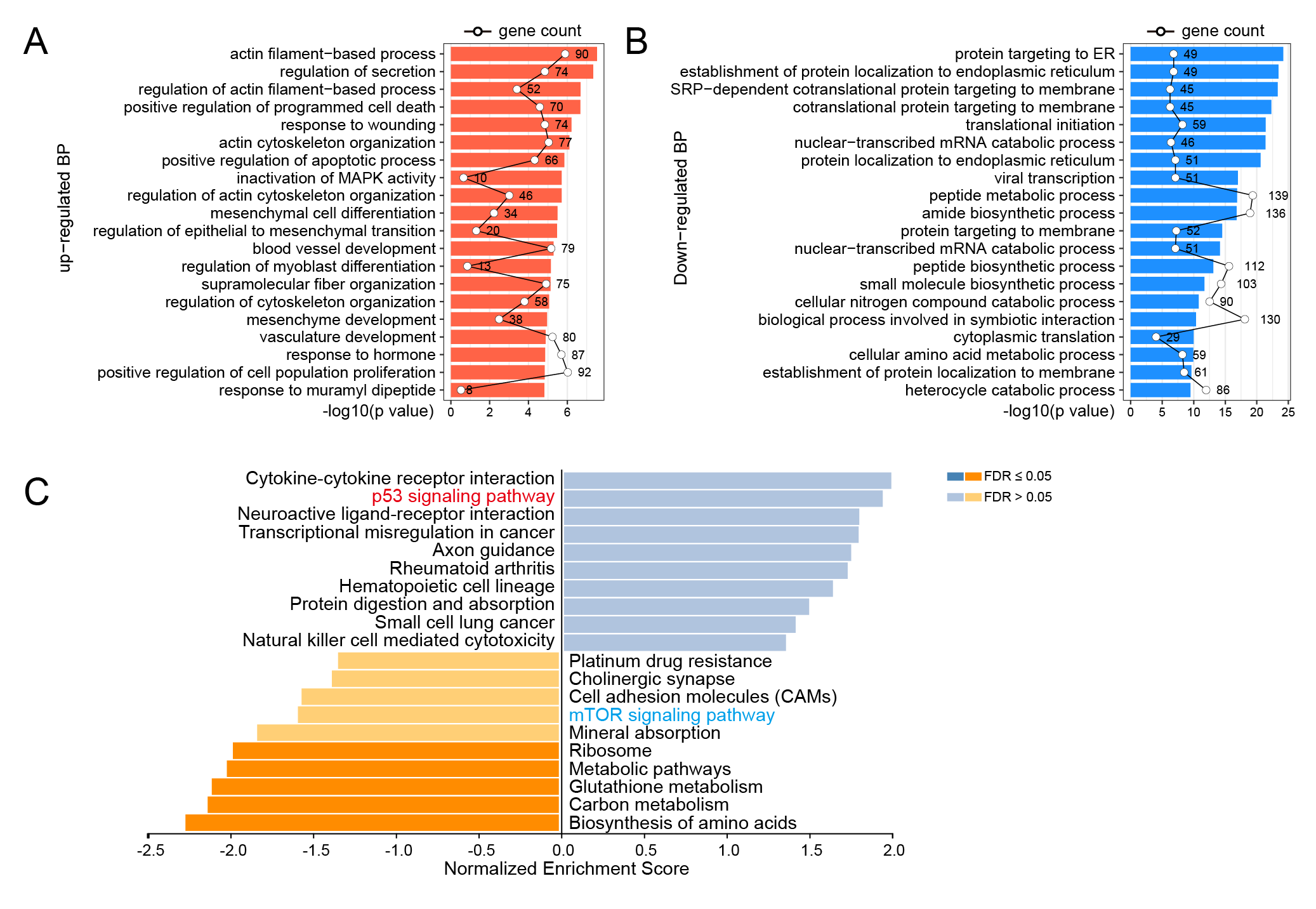

Supplement: Supplementary file 6 [file Image2.TIF]

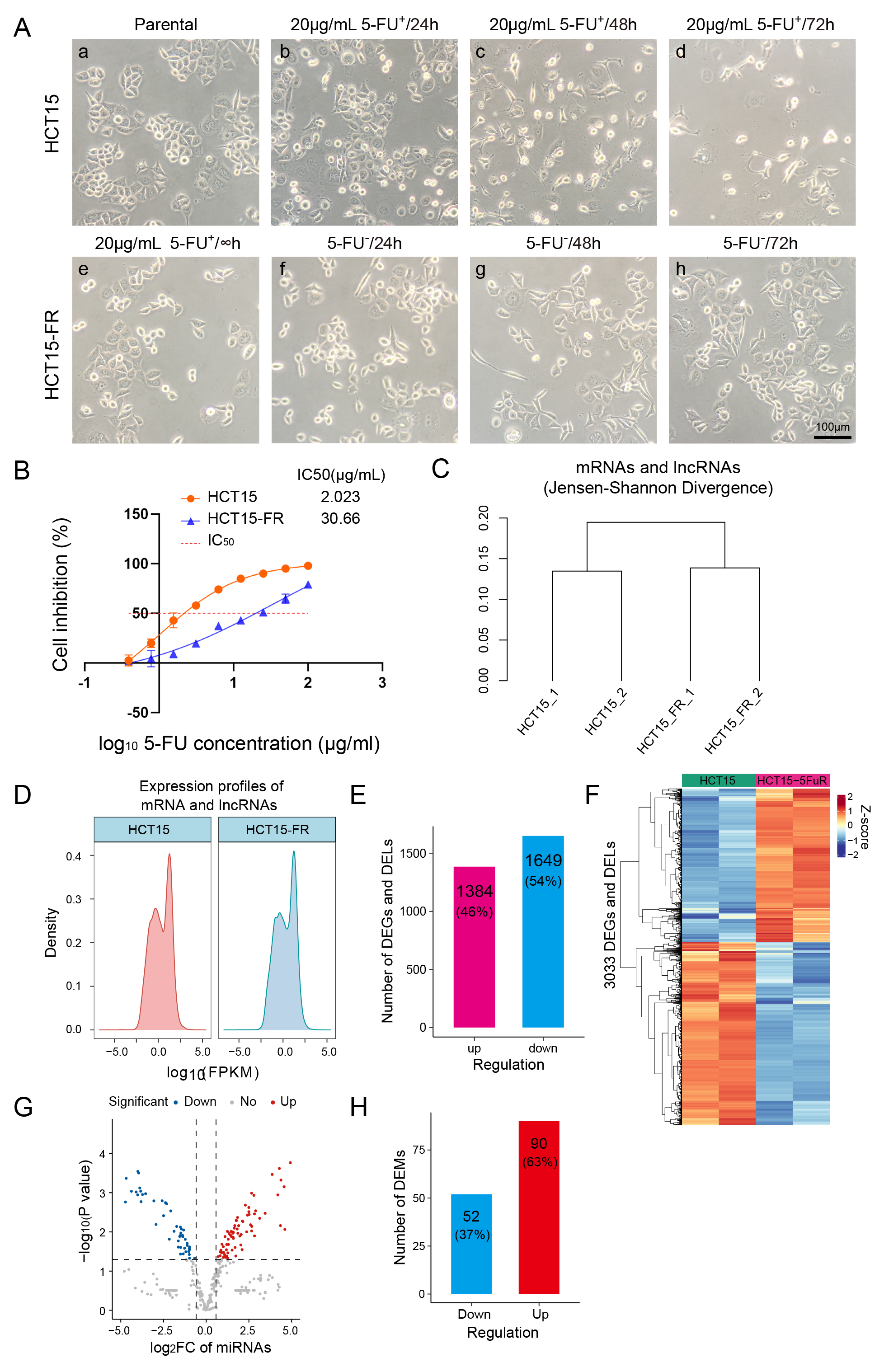

Supplement: Supplementary file 7 [file Image1.TIF]

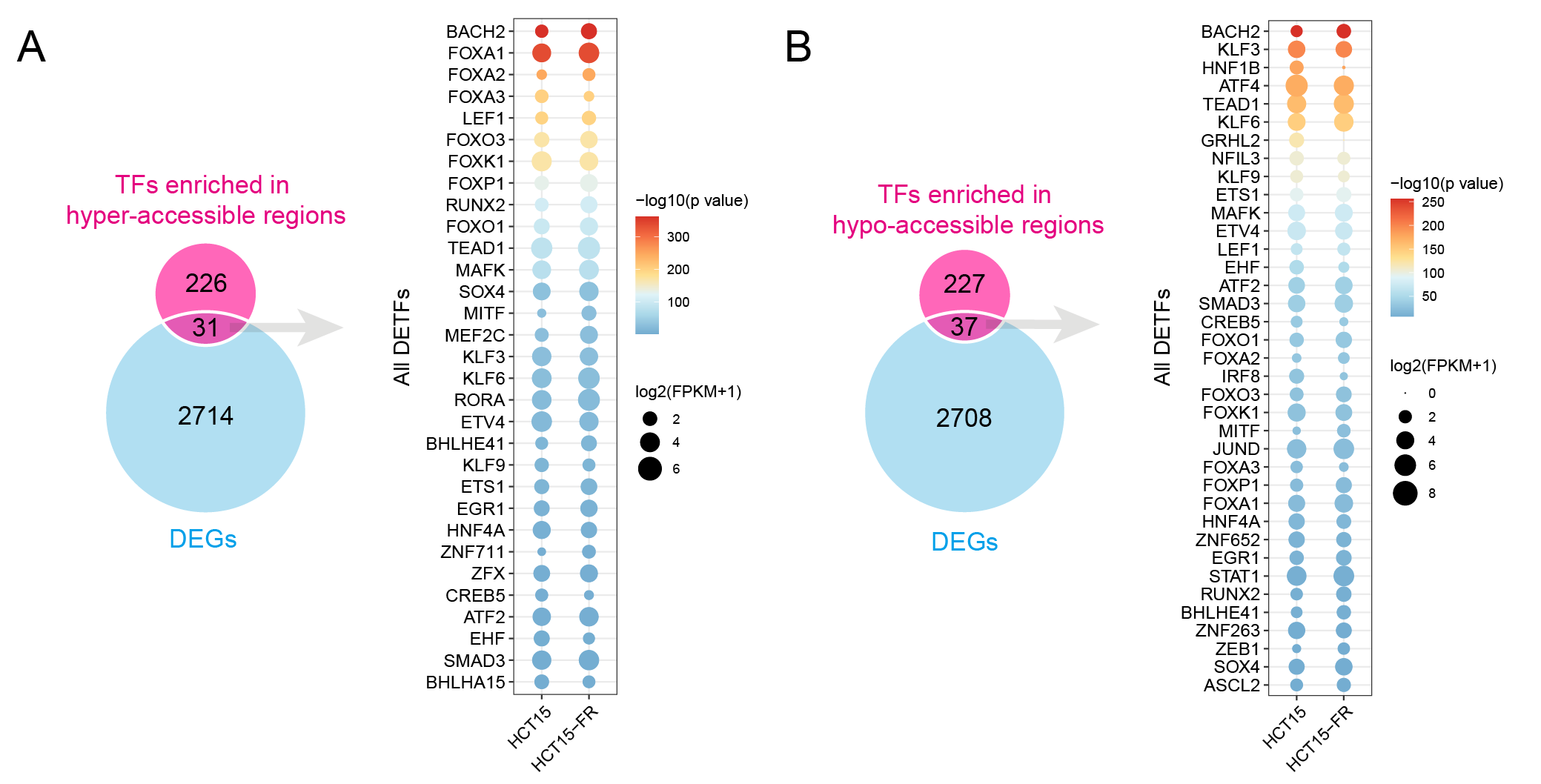

Supplement: Supplementary file 11 [file Image5.TIF]
