# Supplementary material for: Integrated Chromatin Accessibility and Transcriptome Landscapes of 5-Fluorouracil-Resistant Colon Cancer Cells
Source: Front Cell Dev Biol. 2022 Feb 17;10:838332. doi: 10.3389/fcell.2022.838332 (PMC8891516; doi:10.3389/fcell.2022.838332)
Supplement: Supplementary file 14 [file DataSheet1.docx]

Supplementary Material

## Supplementary Figures


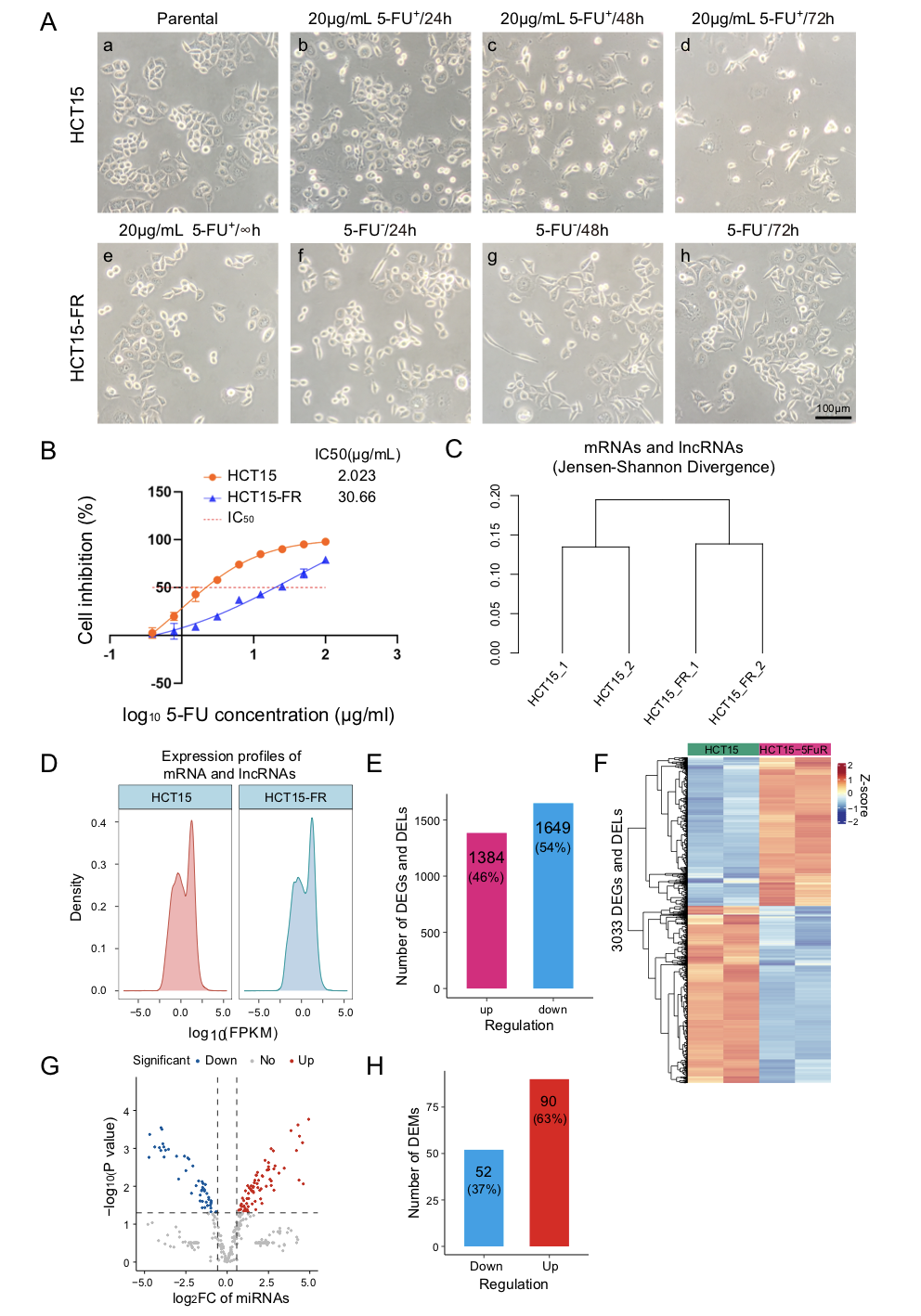


**Supplementary Figure S1.** **Differentially expressed transcripts in 5-FU-resistant HCT15 (HCT15-FR) compared to parental cells.**

(A) (a) showing the morphology of parental HCT15 cells cultured in the drug-free medium; (b-d) represent the HCT15 cells treated with 20 μg/mL 5-FU (5-FU^+^) for 24h, 48h and 72h; (e) indicates the morphology of 5-FU-resistant HCT15 (HCT15-FR) cells cultured in medium with 20 μg/mL 5-FU (5-FU^+^) for more than several months (∞h); (f-h) showing the HCT15-FR cells cultured in the medium without 5-FU (5-FU^-^) for 24h, 48h and 72h. For all further experiments of this study, the HCT15 cells under condition (a) and the HCT15-FR cells under condition (h) were used as the control group and experimental group, respectively. Scale bar, 100 µm.

(B) Growth curve was drawn to evaluate inhibition of cell growth by 5-FU using MTT cell proliferation assay. The 5-FU concentration that results in 50% growth inhibition (IC_50_) for parental and resistant HCT15 cells was shown.

(C) The expression levels (FPKM values) across all mRNA and lncRNA transcripts (hg19 reference genome) were used to calculate Jensen-Shannon Divergence (JSD).

(D) The average gene expression profiles of two biological replicates for each cell line.

(E) Bar plot showing the number and percentage of significantly 1384 up- and 1649 down-regulated mRNAs and lncRNAs between the parental and 5-FU-resistant HCT15 cells.

(F) Heatmap showing the expression levels of 3033 mRNAs and lncRNAs that differentially expressed between HCT15 and HCT15-FR cells. The differentially expressed mRNAs (DEGs) and lnRNAs (DELs) filtered by *P* value < 0.05, fold change (FC) > 1.5 and average FPKM > 3 at least one group. The numeric values were Z-score.

(G) Volcano plot showing fold change and *P* values of all miRNAs. Significantly up-regulated 90 miRNAs identified by DESeq2 were shown as red dots, whereas the 50 down-regulated miRNAs were shown as blue dots.

(H) Bar plot showing the number and percentage of significantly 90 up- and 52 sdown-regulated miRNAs in HCT15-FR compared with the parental cells.

**
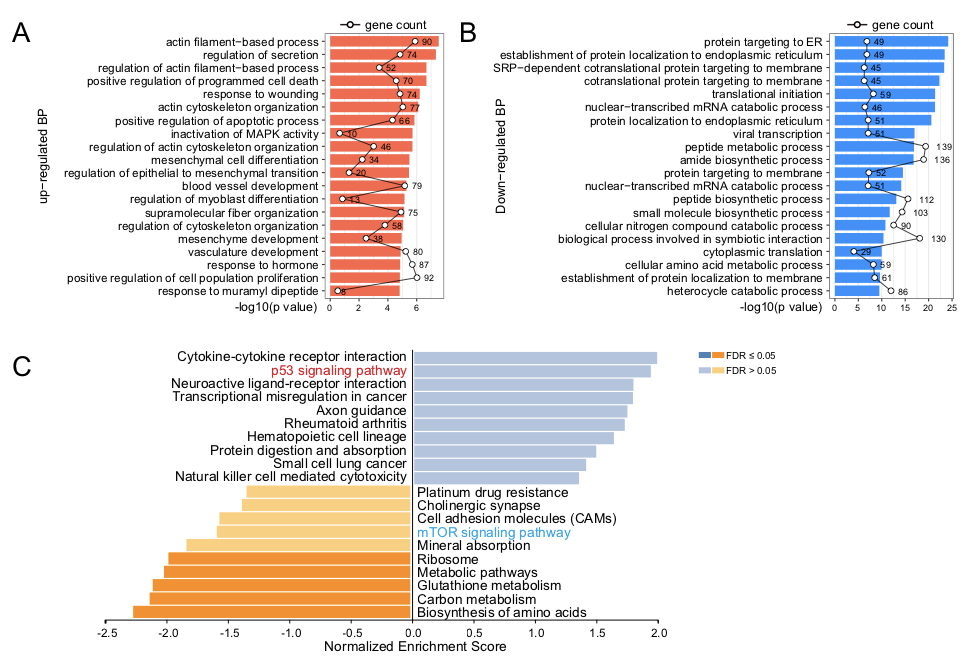
**

**Supplementary Figure S2.** **Functional enrichment analysis for DEGs in HCT15-FR cells.**

(A and B) Top 20 GO based biological process (BP) related to up- (A) and down-regulated (B) genes. The polygonal chain in black shows the count of DEGs enriched in each BP term.

(C) Gene set enrichment analysis (GSEA) using “WebGestalt” for DEG lists ranked by the log_2_(FC). Normalized enrichment score (NES) and FDR were calculated.


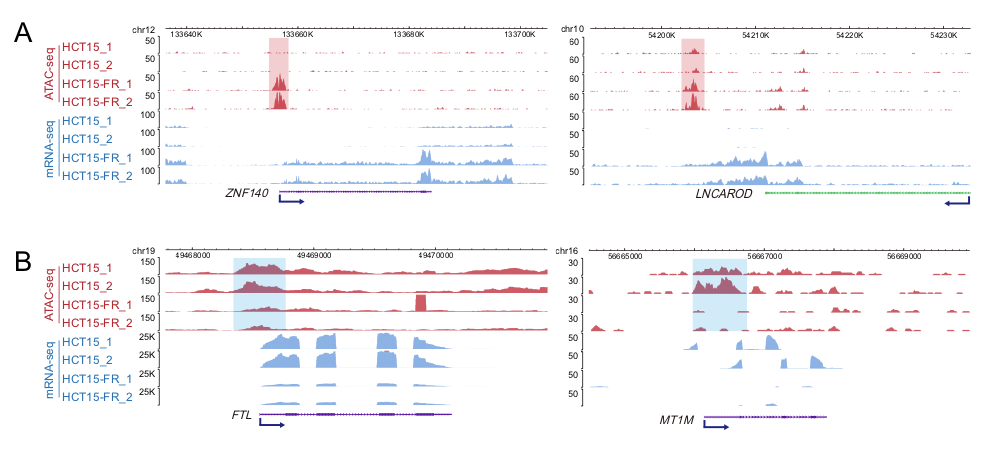
**Supplementary Figure S3.** **DEGs associated with key signal pathway and positively correlated to DARs.**

(A and B) The WashU Epigenome Browser views show ATAC-seq (red) and mRNA-seq (blue) signal of representative up-regulated (A) and down-regulated (B) DEGs associated with KEGG signal pathways (in Figure 4A) and related to hyper- (red shade) and hypo-accessible (blue shade) regions, respectively.


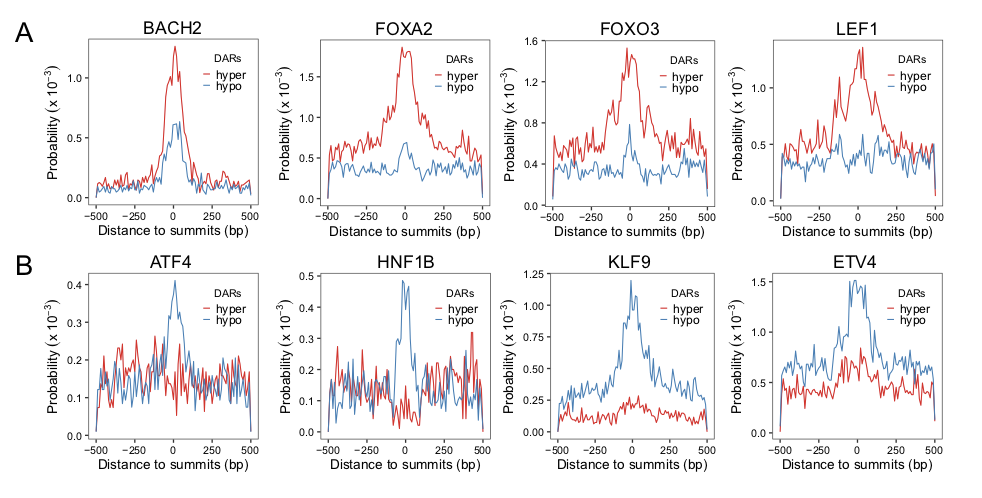


**Supplementary Figure S4. The probability of DETF occupancy at DARs.**

(A and B) Distribution probability of TF-binding motifs surrounding (from -500bp to +500bp) ATAC-seq peak summits located in hyper- (A) and hypo-accessible (B) regions.


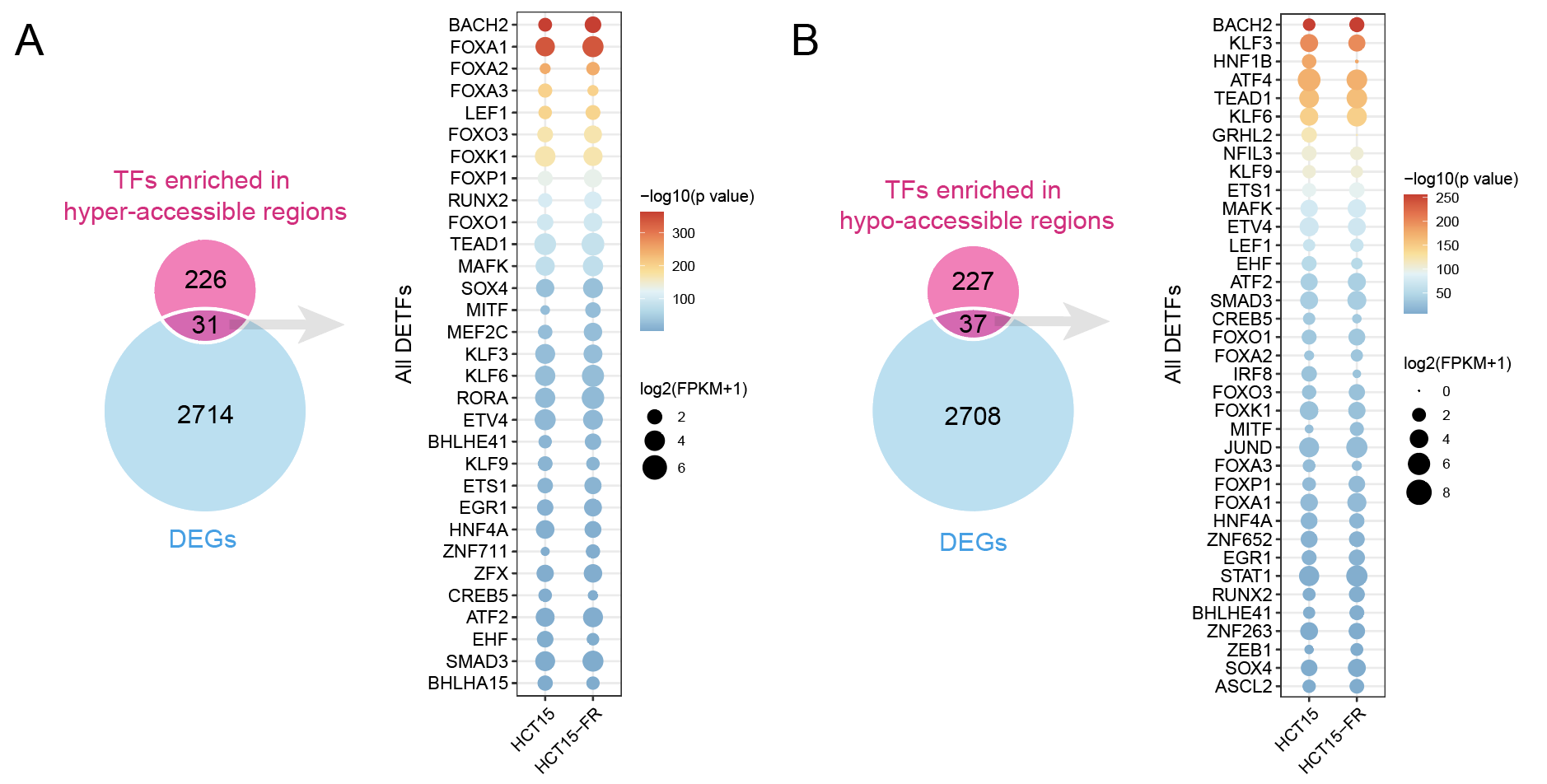


**Supplementary Figure S5. Identification of DETFs associated with DARs.**

(A) Venn diagram showing overlap of the corresponding TFs for each motif in hyper-accessible regions and DEGs. The dot plot indicates the identified 31 DETFs. The size of each dot represents the mRNA expression level of the enriched motif cognate TFs. Only TFs motif enrichment *P*-value < 0.05 were included. The color of each dot represents different *P*-values for enriched motifs.

(B) Venn diagram showing overlap of the corresponding TFs for each motif in hypo-accessible regions and DEGs. The dot plot indicates the identified 37 DETFs.


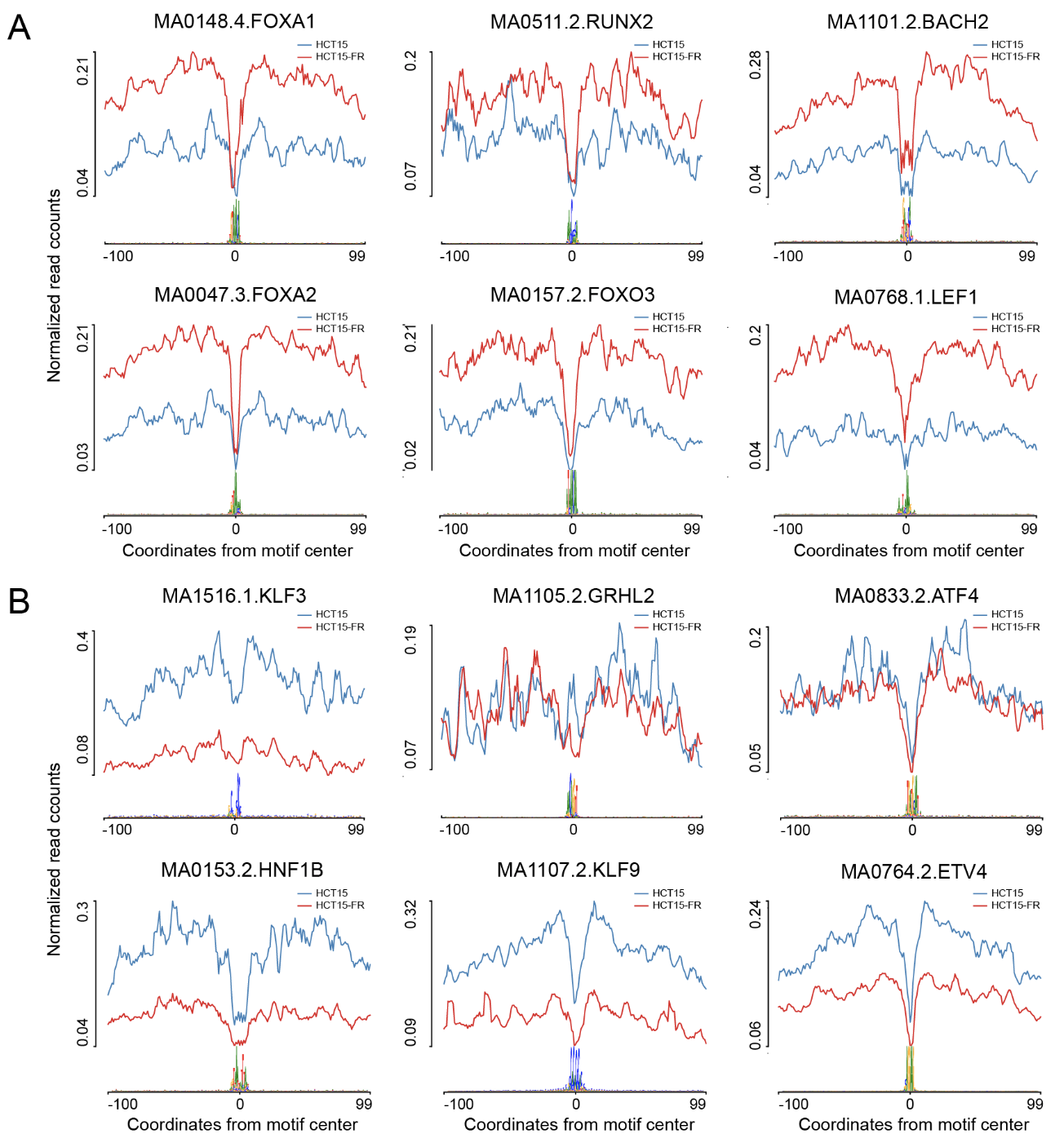


**Supplementary Figure S6. The footprint profiles of particular DETF associated with DARs.**

(A and B) Average ATAC-seq profiles around binding sites of up- (A) and down-regulated (B) TFs (Figure 7C and S4) associated with hyper- (A) and hypo-accessible (B) regions in parental and 5-FU resistant HCT15 cells.

## Supplementary Tables

**Supplementary Table S1. FPKM of all mRNAs and lncRNAs.**

**Supplementary Table S2. FPKM of differentially expressed mRNAs and lncRNAs.**

**Supplementary Table S3. Read count of all miRNAs.**

**Supplementary Table S4. Differentially expressed miRNAs identified by DESeq2.**

**Supplementary Table S5. Annotation of ATAC-seq peaks identified by MACS2.**

**Supplementary Table S6. Annotation of differentially accessible regions (DARs).**

**Supplementary Table S7. Known TF binding motifs enriched in differentially accessible regions (DARs).**
